# Therapeutic reprogramming of tumour-associated macrophages in pancreatic cancer using a cytotoxic CCR2-targeted nanotheranostic

**DOI:** 10.1186/s12943-026-02597-7

**Published:** 2026-02-07

**Authors:** Vikas Kumar Somani, Xiaohui Zhang, Timothy Hung-Po Chen, Ashenafi Bulle, Sapana Bansod, Lin Li, Yutong Geng, Liang-I Kang, Gyu Seong Heo, Hannah Luehmann, Yuena Zhang, Muhammad A. Saeed, Kory J. Lavine, David G. DeNardo, Russell K. Pachynski, Yongjian Liu, Kian-Huat Lim

**Affiliations:** 1 Department of Internal Medicine, Division of Oncology, Washington University School of Medicine, 660 South Euclid Avenue, Campus Box 8069, St. Louis, Missouri 63110 United States; 2 Department of Radiology, Washington University School of Medicine, St. Louis, Missouri United States; 3 Department of Internal Medicine, Division of Cardiology, Washington University School of Medicine, St. Louis, Missouri United States

## Abstract

**Background:**

Pancreatic ductal adenocarcinoma (PDAC) exhibits a profoundly immunosuppressive tumour microenvironment (TME) dominated by inflammatory monocytes (IMs) and tumour-associated macrophages (TAMs), which restrict adaptive immunity and drive resistance to immune checkpoint blockade (ICB). Recruitment of CCR2⁺ IMs by tumour-derived CCL2 is a central mechanism underlying TAM accumulation. Conventional gemcitabine (GEM) and small-molecule CCR2 inhibitors provide limited benefit due to poor intratumoural delivery, transient target engagement, and compensatory myeloid recruitment.

**Methods:**

We engineered a CCR2-targeted nanotheranostic by conjugating a CCR2-binding peptide (ECL1i) and GEM onto ultrasmall copper nanoclusters (CuNCs-ECL1i-GEM; C-E-G). Therapeutic efficacy and immune remodelling were evaluated using orthotopic subcutaneous and the autochthonous PDAC mouse models model, using scRNAseq, flow cytometry, multiplex immunohistochemistry, and in vitro functional assays.

**Results:**

C-E-G exhibited robust tumour accumulation and selectively eliminated CCR2⁺ TAMs without systemic myelotoxicity, while durably reprogramming residual macrophages. Mechanistically, C-E-G induced the emergence of an immunostimulatory CCRL2⁺ TAM subset through true macrophage repolarization rather than monocyte replacement. CCRL2⁺ TAMs accumulated intratumoural chemerin, upregulated antigen-presentation and co-stimulatory programs, and were essential for CD8⁺ T-cell recruitment and activation. Genetic and orthotopic studies confirmed their CCR2-independent origin and requirement for tumour control. C-E-G remodelled the TME toward a lymphocyte-permissive inflammatory state and synergized with ICB to induce complete tumour regression and prolong survival in KPPC mice.

**Conclusions:**

CCR2-targeted cytotoxic nanotherapy eliminates immunosuppressive CCR2 + TAMs, reprograms the macrophage landscape including CCRL2⁺ TAMs, and unlocks durable anti-tumour immunity in PDAC, supporting translational development of this strategy.

**Supplementary Information:**

The online version contains supplementary material available at 10.1186/s12943-026-02597-7.

## Introduction

Immune checkpoint blockade (ICB) remains largely unsuccessful in the treatment of pancreatic ductal adenocarcinoma (PDAC), largely due to the profound immunosuppressive tumour microenvironment (TME) dominated by the myeloid-derived suppressor cells (MDSCs) such as inflammatory monocytes (IMs) and tumour-associated macrophages (TAMs) [1, 2]. Serial preclinical work showed that IMs expressing C-C chemokine receptor type 2 (CCR2) are chemotactically drawn from the bone marrow by the high C-C motif chemokine ligand 2 (CCL2) in the PDAC TME, where they subsequently evolve into TAMs [1, 3].

We recently completed a phase 1 clinical trial combining chemotherapy with BMS813160, a CCR2/5 inhibitor, and nivolumab for patients with borderline resectable or locally advanced PDAC, with the aim of improving surgical resection [4]. While this study was not powered for efficacy and survival, the objective response rate (ORR) was promising compared to historical data. Analysis of the pre- and post-treatment tumours treated with the quadruplet therapy showed decreased intratumoural monocytes, macrophages, enhanced T cell proliferation and effector gene expression. However, for both clinical trials, > 80% of patients with locally advanced PDAC did not achieve sufficient tumour downstaging to proceed with surgical resection. We speculate that a reason for this limited efficacy is that small molecule CCR2 inhibitors like BMS-813,160 are reversible inhibitors and thus can only temporarily block CCR2 receptors on IMs, leading to the transient “trapping” of these cells in the bone marrow [5]. Furthermore, CCR2i could cause compensatory upregulation of circulating CCL2 levels in both human and mice [6]. As such, when the serum concentration of CCR2is drops below a certain threshold, these “trapped” IMs may still enter the circulation and infiltrate the PDAC TME. Therefore, a CCR2-targeted agent that is more durable and can eliminate, instead of sequestering the CCR2^+^ cells, should lead to better response when combined with ICB.

In this study, we develop a potent CCR2-targeted therapeutic by conjugating a CCR2-binding peptide (ECL1i) and cytotoxic chemotherapeutic, gemcitabine (GEM) onto the surface of ultrasmall biodegradable copper nanoclusters (CuNCs-ECL1i-GEM, or C-E-G). We demonstrate that C-E-G efficiently delivers GEM into CCR2-expressing cells, reduces intratumoural TAMs in PDAC tissues and reprograms TAMs that render ICB effective.

## Results

### Development of a cytotoxic CCR2-Targeted nanotheranostic

From analysis GEPIA2 which contains The Cancer Genome Atlas (TCGA) dataset, PDAC displays higher *CCR2* expression than normal pancreatic tissues and most other cancer types, especially with in more advanced stages. *CCL2* is also more highly expressed in PDAC, an interestingly PDAC displayed the highest positive correlation between these two genes (Suppl. Fig. S1a-e), supporting a pathogenic role of the CCR2-CCL2 axis in PDAC biology.

**Fig. 1:**
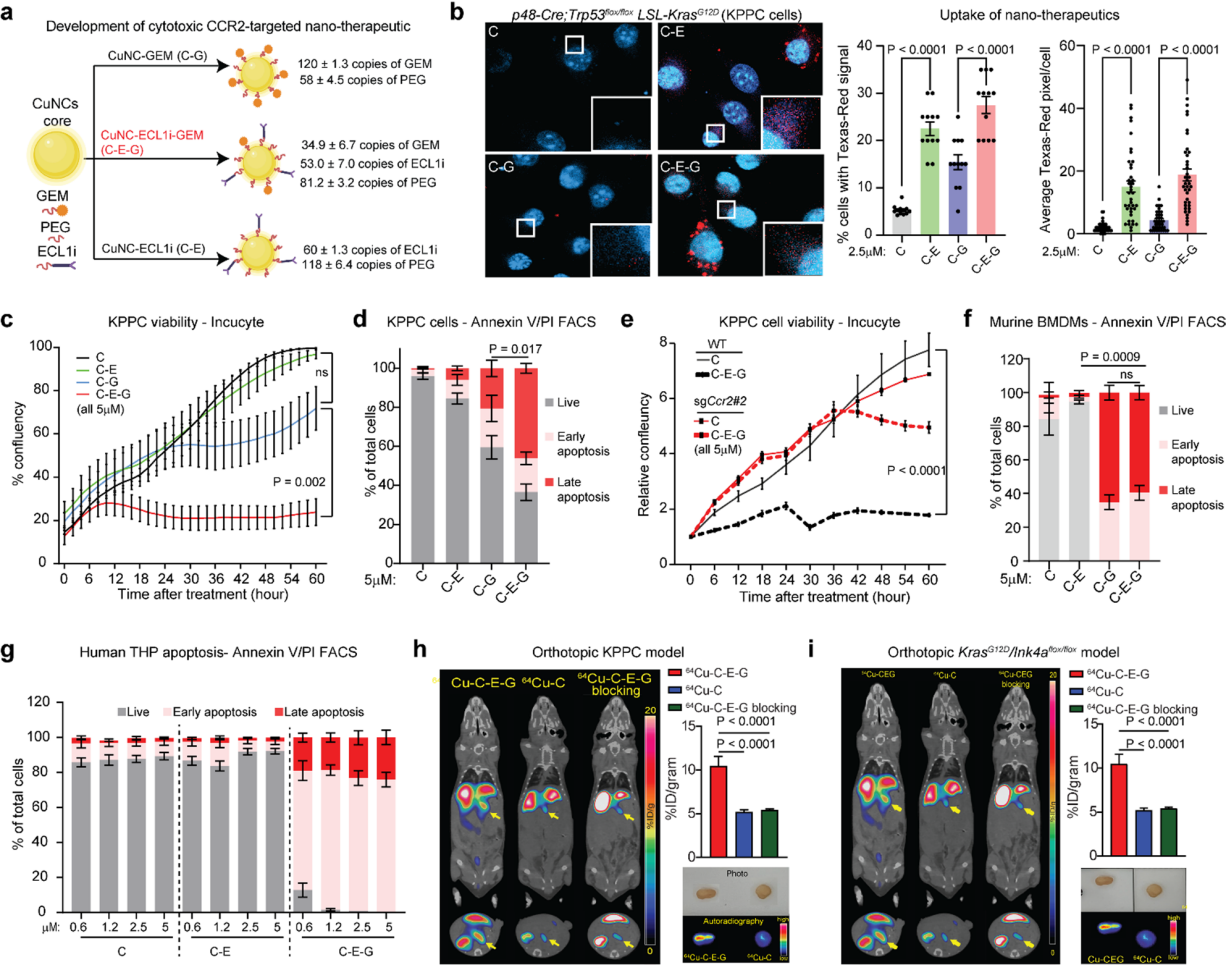
Development of a Cytotoxic CCR2-Targeted Nanotheranostic. **a** Schematic illustrating the development of different copper-based CCR2-targeted nanotheranostic used in this study. **b** Representative IF images and quantification of binding of the indicated Texas Red -labelled nanotheranostic on KPPC cells at a concentration of 2.5 µM after 12 h treatment. **c** Viability of KPPC cells treated with the indicated nanotheranostics at 5µM, as measured periodically by cell confluency using Incucyte® live-cell imager. **d** Quantification of apoptotic KPPC cells treated with the indicated nanotheranostics at 5 µM for 48 h followed by FACS using Annexin V and PI staining. Early apoptosis: Annexin V positive/PI negative; late apoptosis: double positive. **e** Relative viability of control or Ccr2-ablated KPPC cells treated with C or C-E-G at 5 µM as measured by cell confluency using periodic Incucyte imaging. Quantification of apoptosis of (**f**) murine BMDMs or (**g**) human THP-1 monocyte cells treated with the indicated nanotheranostics for 48 h followed by FACS using Annexin V and PI staining. PET images and quantification of 64Cu-labelled C or C-E-G signals in the orthotopic (**h**) KPPC or (**i**) KI tumours in live tumour-bearing mice. Competitive blocking using non-radiolabelled C-E-G was used to quench the signal of 64Cu-C-E-G. Right lower corners: ex vivo autoradiography of KPPC or KI tumours harvested 24 h after mice were treated with 64Cu-C-E-G. All quantitative data were presented as means ± SEM and analyzed using one- or two-way ANOVA with Welch or Tukey’s multi-comparison tests (***P < 0.001, **P < 0.01)

We previously identified ECL1i peptide as a specific ligand that binds CCR2^+^ cells with high affinity and can be conjugated with ^64^Cu to enable in vivo imaging in PDAC bearing mice [7, 8]. On this basis, we synthesized bidentate thioctic acid (TA) based ligands including TA-poly(ethylene glycol) (PEG, MW = 750 Da), TA-PEG-ECL1i, TA-PEG-GEM and conjugated them to the surface of copper nanocluster (CuNC, termed C onwards) to produce CuNC-ECL1i (C-E), CuNC-GEM (C-G) or CuNC-ECL1i-GEM (C-E-G). Mass spectrometry showed each C-E-G molecule contained approximately 1000 Cu atoms, which enable a surface conjugation of ~ 180 TA-based ligands, offering the flexibility to optimize the TA-PEG : TA-PEG-ECL1i : TA-PEG-GEM ratio for favourable pharmacokinetics and maximal targeting efficiency [7, 8] (Fig. 1a). To test the binding specificity of C-E-G, we treated a murine PDAC cell line derived from a *p48-Cre*,* Trp53*^*flox/flox*^, *LSL-KRas*^*G12D*^ (KPPC) mouse with C, C-E, C-G or C-E-G labelled with Texas-Red dye and performed fluorescence microscopy. We found significantly higher Texas-Red signals in KPPC cells treated with C-E, or C-E-G (Fig. 1b), confirming the binding specificity of ECL1i. We previously showed that the biodegradability of our CuNC and its TA-based ligands was pH sensitive with ∼55% degradation within 4 h (h) at pH 5.5 while only 10% at pH 7.4. This unique feature allows rapid release of TA-PEG-GEM within the acidic lysosomes (pH 5.5 - pH 6.8) of PDAC cells [8]. As such, C-E-G, but not C or C-E, and to a lesser extent C-G, stalled the growth of KPPC cells, as measured by cell confluency over time using Incucyte^®^ live cell imager (Fig. 1c). At 5 mM, C-E-G induced more apoptosis than C-G, as illustrated by Annexin V staining by flow cytometry (FACS, Fig. 1d). As further confirmation of specificity, C-E-G did not inhibit the growth of KPPC cells in which the *CCR2* gene was ablated with CRISPR/Cas9 (Suppl. Fig.S1f, Fig. 1E).

Because we developed C-E-G to specifically target IMs and TAMs, we tested its suppressive effect on murine bone marrow derived macrophages (BMDMs). Strikingly, C-E-G at 5 µM resulted in early or late apoptosis in almost all BMDMs compared to C or C-E, while C-G also had similar pro-apoptotic effect reasonably due to the direct exposure to gemcitabine (Fig. 1f). In human monocytic cell line THP-1 known to express CCR2 [9], treatment with C-E-G as low as 0.6 mM overnight resulted in > 80% early and late apoptosis (Fig. 1g). To ensure that C-E-G can accurately target PDAC tumours in vivo, we inoculated KPPC or *KRas*^*G12D*^*/Ink4a*^*flox/flox*^ (KI) cells, a PDAC cell line derived from a *KRas*^*G12D*^*/Ink4a*^*flox/flox*^ PDAC mouse, into the pancreas of syngeneic C57BL/6J or FVBN/J mice, respectively. Two weeks after inoculation, we performed positron emission tomography/computed tomography (PET/CT) imaging using ^64^Cu-radiolabelled CuNC (^64^Cu-C), or C-E-G (^64^Cu-C-E-G). Despite liver accumulation due to the physicochemical properties of CuNCs [8], ^64^Cu-C-E-G demonstrated intense accumulation within the orthotopic tumours, in contrast to the low tumour uptake of the non-targeted ^64^Cu-C. PET signal emanated from ^64^Cu-C-E-G was quenched in mice co-treated with non-radioactive C-E-G as a competitive blockade. Correspondingly, both KPPC and KI tumours labelled with ^64^Cu-C-E-G emitted stronger signals by ex vivo autoradiography compared with ^64^Cu-C-treated tumours (Fig. 1h and i). Together, C-E-G demonstrated specificity towards CCR2 and has the potential to deliver gemcitabine into CCR2^+^ monocytes and macrophages in vivo.

### C-E-G promotes a T cell-permissive TME in autochthonous KPPC tumours

**Fig. 2:**
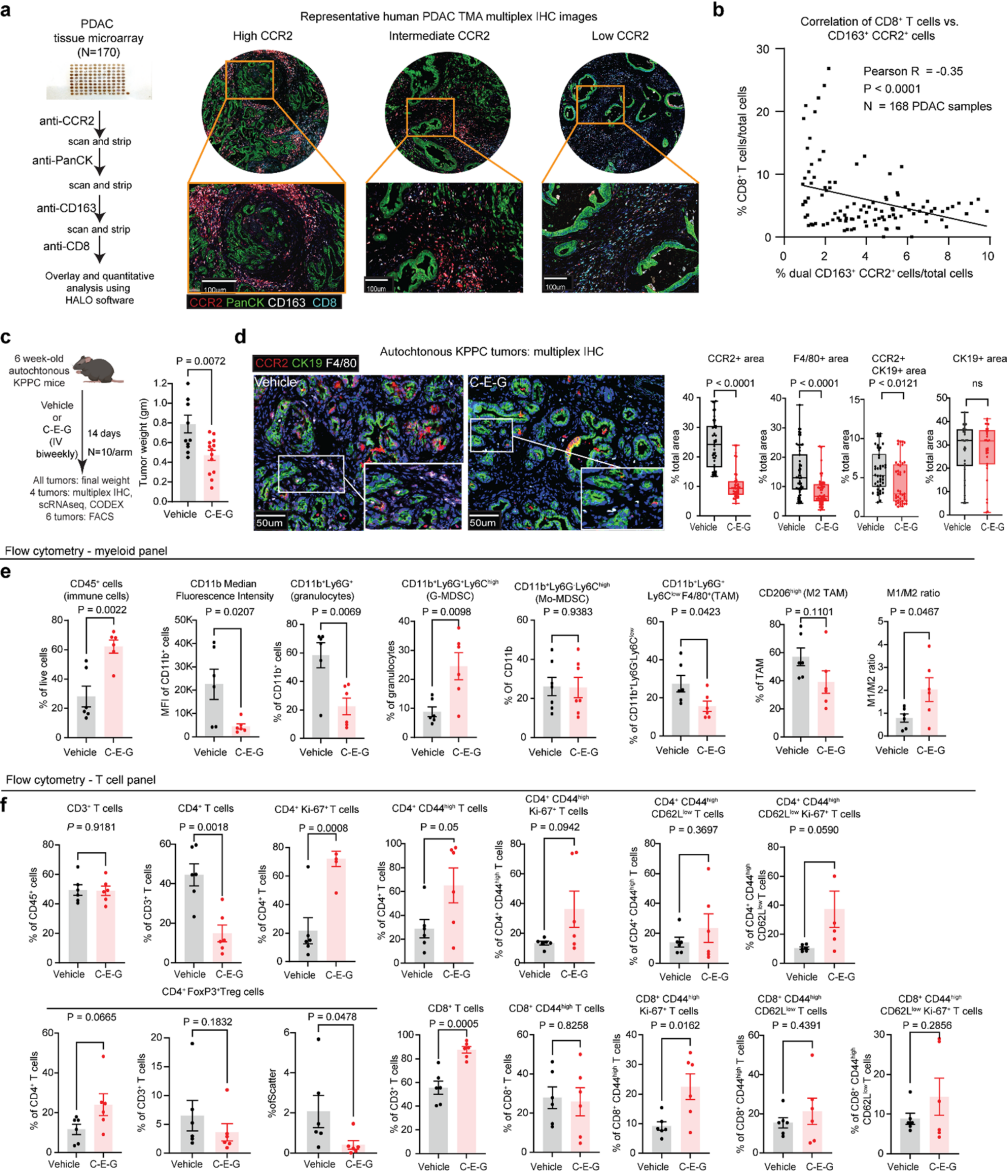
C-E-G promotes a T cell-permissive TME in autochthonous KPPC tumours. **a** Schema illustrating the workflow of multiplex IHC on a human PDAC TMA containing 168 separate PDAC samples and representative images of three PDAC samples representing different staining intensities of CCR2, as measured using HALO® imaging software. The antibodies used were previously optimized and published [32]. **b** Scatter plot showing the negative correlation by Pearson coefficient between the abundance of CD8+ T cells and dual CD163+/CCR2+ cells (both per total cells by DAPI) across different samples in the PDAC TMA. **c** Experimental schematics of autochthonous KPPC mice treated with vehicle (PBS) or C-E-G intravenously biweekly and the final tumour weights. **d** Representative multiplex IHC images and quantification of CCR2, F4/80 and CK19 areas between vehicle and C-E-G-treated KPPC tumours. 6tumours/arm were analyzed, and 5–6 fields covering the entire tumour were quantified using HALO® software. FACS-based quantification of the indicated intratumoural (**e**) myeloid and (**f**) T cells from autochthonous KPPC mice (*N* = 6). *P* values calculated by two-tailed unpaired t-test; scale bars = 50 μm)

Studies in mouse models have demonstrated that targeting CCR2^+^ myeloid cells can reinvigorate cytotoxic CD8^+^ T cells [10]. However, the correlation between the abundance of CCR2^+^ myeloid and CD8^+^ T cells in human PDAC has not been reported. To address this, we performed multiplex immunofluorescence staining (IF) analyses by sequential antibody staining on a PDAC tissue microarray (TMA) comprising 168 chemotherapy-naïve, surgically resected PDAC tumours (Fig. 2a). We observed significant inverse correlation between the abundance of intratumoural CCR2^+^ CD163^+^TAMs and CD8^+^ T cells (Pearson coefficient = -0.35, *P* < 0.0001, Fig. 2b), providing the first evidence that the CCR2^+^ TAMs is associated with CD8^+^ T cell exclusion in human PDAC.

Next, we evaluated the effects of C-E-G in autochthonous KPPC mice, a highly aggressive mouse model known to develop desmoplastic, myeloid-rich PDAC [11]. At 6 weeks of age, ^64^Cu-C-E-G PET revealed a significantly higher tumour uptake than ^64^Cu-C in the KPPC mice. (Suppl. Fig.S2a, S2b). Moreover, CCR2 was expressed on both TAMs (F4/80^+^), and neoplastic cells (CK19^+^, Suppl. Fig.S2c), albeit at < 10%. We treated 6-week-old KPPC mice, with intravenous C-E-G injections twice per week for two weeks, a duration adequate to discern immunologic changes before the mice succumb to advanced PDAC [11]. Tumours were harvested four days after completion of the final (fourth) treatment for analysis. Compared to vehicle controls, C-E-G–treated PDAC tumours were significantly smaller by weight (Fig. 2c) and had markedly decreased CCR2^+^ and F4/80^+^ areas, whereas the CK19^+^, aSMA and Sirius red areas remained unchanged (Fig. 2d and e, Suppl. Fig. S2c, S2d). Correspondingly, cultured BMDMs were significantly more sensitive to the suppressive effect of C-E-G than KPPC cells or cancer-associated fibroblasts (CAFs) isolated from KPPC tumours (Suppl. Fig. S2e). These data suggest that although C-E-G binds to both PDAC cells and macrophages, the primary in vivo target is CCR2-expressing macrophages due to their higher susceptibility to gemcitabine.

Next, we performed flow cytometry (FACS) to discern the immunologic changes. Compared to controls, C-E-G-treated tumours had more total CD45^+^ immune cells, less CD11b-expressing myeloid cells, granulocytes and TAMs, which were shifted towards a more M1-polarized phenotype (CD206^low^, Fig. 2e), less total CD4^+^ T cells, which were more proliferative (Ki-67^+^) and trended towards an effector phenotype (CD44^high^); as well as more total and proliferating effector CD8^+^ T cells (CD44^high^, Ki-67^+^, Fig. 2f). These data demonstrate the immunomodulatory potential of C-E-G in reducing immunosuppressive TAMs and revitalizing adaptive T cell immunity in PDAC.

### C-E-G reprograms TAMs towards an inflammatory phenotype

**Fig. 3:**
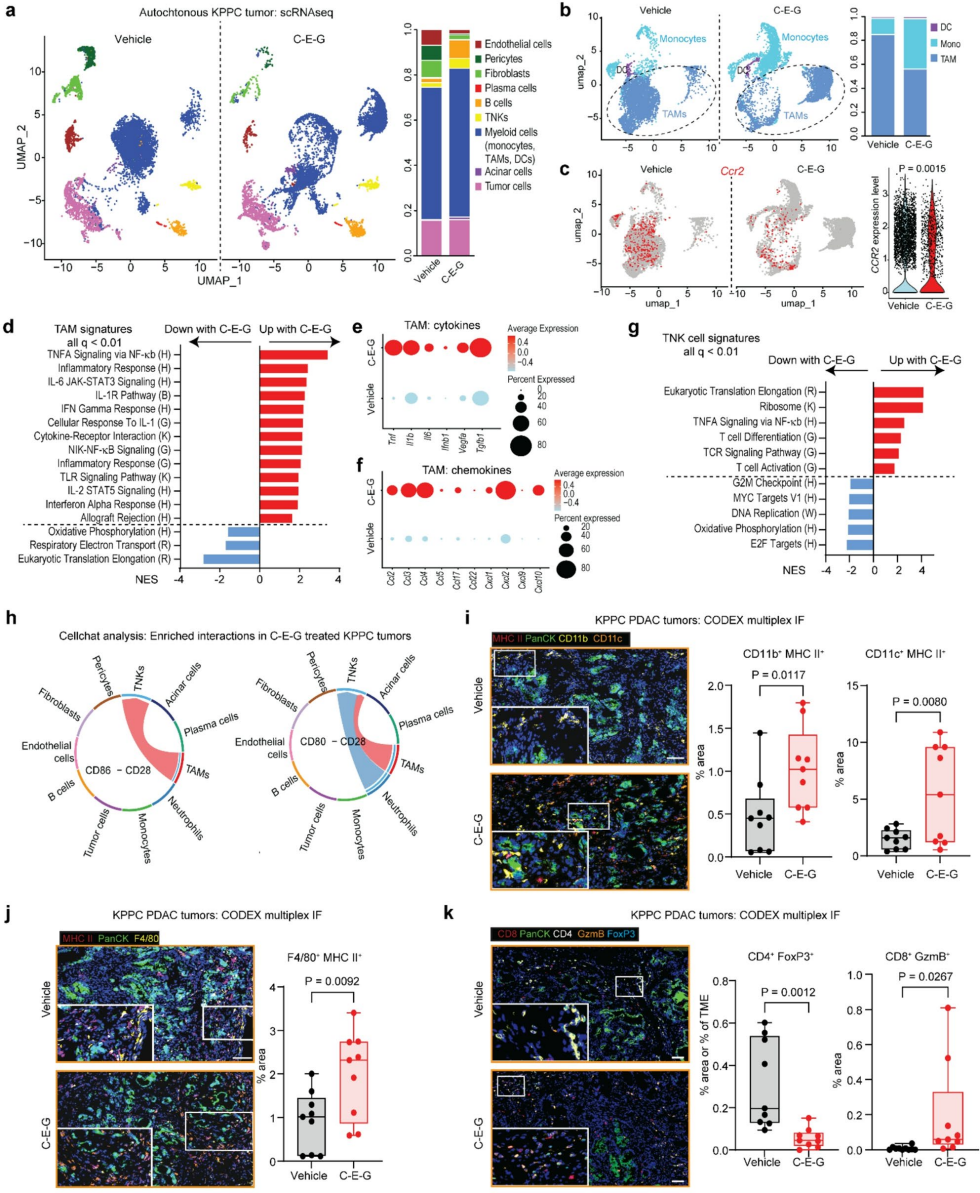
C-E-G reprograms TAMs towards an inflammatory phenotype. UMAP plots and bar graphs depicting changes in relative abundance of (**a**) all different cell subsets, (**b**) myeloid cell subsets and (**c**) CCR2-expressing myeloid cells in autochthonous KPPC tumours treated with vehicle or C-E-G for 2 weeks. (**d**) Bar graph showing the enrichment of selected pathway signatures in intratumoural TAMs from vehicle- or C-E-G-treated KPPC tumours. Dot plots showing statistically significant upregulation of expression of (**e**) cytokine and (**f**) chemokine genes in intratumoural TAMs from vehicle- or C-E-G-treated KPPC tumours. (**g**) Bar graph showing the enrichment of selected pathway signatures in intratumoural T and NK cells from vehicle- or C-E-G-treated KPPC tumours. (**h**) Cellchat analysis nominating pathway interaction between TAMs and TNK cells in C-E-G-treated tumours. Representative multiplex IHC images and quantification of (**i**) CD11b+, CD11c + or (**j**) F4/80 cells that were also positive for MHC II staining in vehicle- or C-E-G-treated KPPC tumours. (Scale = 100 μm) (**k**) Representative multiplex IHC images and quantification of CD4+ FoxP3+ and CD8+ GzmB+ areas in vehicle- or C-E-G-treated KPPC tumours. For (**i**-**k**), 3 tumours/arm were analyzed, and 3 fields/tumour covering the entire tumour/TME (represented as % area) were quantified using HALO® software. *P* values calculated by two-tailed unpaired t-test; scale bars = 50 μm

To understand the impact of C-E-G in greater details, PDAC from 6-week-old KPPC mice treated with vehicle or C-E-G (7 mg/kg/dose) biweekly for two weeks were subject to single cell (sc)RNAseq four days after the last injection. Uniform manifold approximation and projection (UMAP) analysis showed no significant change in the abundance of PDAC cells, likely due to their relative resistance to gemcitabine at such low dose, and interestingly slight increase in total myeloid cells (Fig. 3a, Suppl. Fig. S3a). However, C-E-G-treated tumours had decreased total and CCR2-expressing TAMs, and relatively more monocytes (Fig. 3b and c), suggesting a block of differentiation from IMs to TAMs. Pathway analysis showed that C-E-G treated TAMs displayed significantly higher levels of inflammatory signatures including the TNFA, IL-1, IL-6, IFNa and IFNg pathways (Fig. 3d), and correspondingly inflammatory cytokines and chemokines including *Tnf*,* Il1b*,* Il6*, *Ccl2*,* Ccl3*,* Ccl4*,* Cxcl2* and *Cxcl10* (Fig. 3e and f, Suppl. Fig. S3b), which could contribute to treatment resistance. Cellchat analysis suggested that TNF produced from C-E-G treated TAMs and neutrophils may engage TNFR1 and 2 in CAFs, tumour, and endothelial cells within the TME (Suppl. Fig. S3c), implying an inflammatory response on multiple cell types in the TME. In support, CAFs in C-E-G treated tumours displayed upregulation of TNF and IFNg signatures, indicative of a shift toward an inflammatory phenotype (Suppl. Fig. S3d). The TNK cells in C-E-G treated tumours also displayed upregulated TNF signature and activated T cell signatures (Fig. 3g). Cellchat analysis showed an increased CD80/86-CD28 interaction (Fig. 3h) between TAMs and TNK cells in C-E-G treated tumours. As CD80 and CD86 are co-stimulatory molecules on antigen-presenting cells that engage CD28 and activate T cells [12, 13], these findings suggest that C-E-G treatment results in TAMs with enhanced antigen-presenting ability to promote T cell response. To confirm these RNAseq data, we performed the Co-detection by indexing (CODEX)-based multiplex IF on the KPPC tumours. Compared to control tumours, C-E-G-treated tumours had higher abundance of F4/80, CD11b and CD11c cells that co-express MHC II (Fig. 3i and j), decreased abundance of CD4^+^ FoxP3^+^ Treg cells and increased abundance of activated CD8^+^ T cells (GzmB^+^, Fig. 3k). These data corroborate with our FACS data and showed that C-E-G shifts TAMs towards a pro-inflammatory/M1 phenotype which may invoke T cell response.

### C-E-G augments antigen presentation by TAMs to activate CD8^+^ T cells

**Fig. 4:**
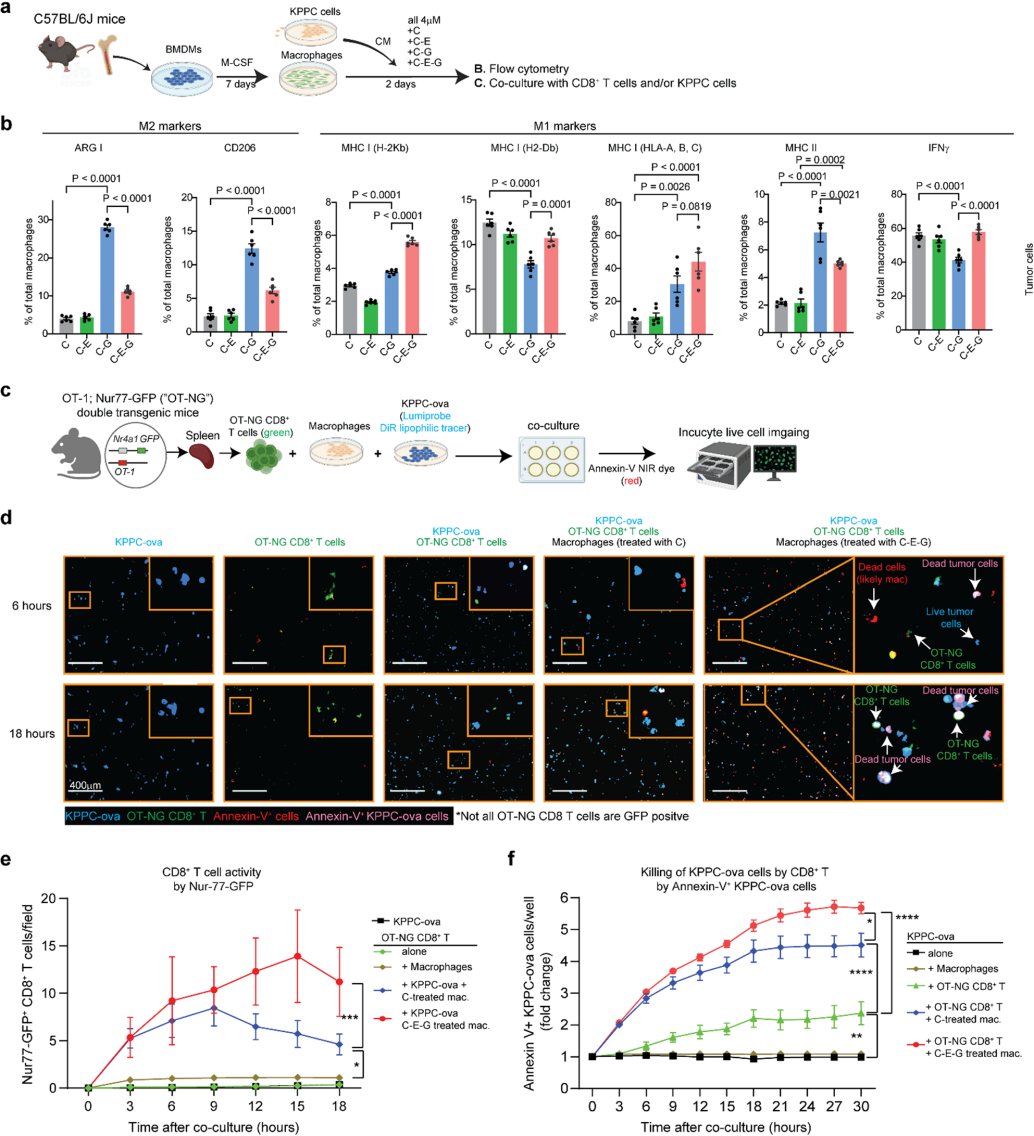
C-E-G augments antigen presentation by TAMs to activate CD8^+^ T cells. **a** Schematic illustrating isolation of murine BMDMs, differentiation into macrophages followed by incubation with conditioned media from cultured KPPC tumour cells and different nanotheranostics at 4 μM for 2 days. The macrophages were then subject to various analyses. **b** FACS showing changes in expression of M2 (Arg1, CD206) and M1 markers (MHC I, MHC II, IFNγ) (**c**) Schematic and representative images showing the co-culture of KPPC-ova or OT-NG CD8^+^T alone or in combinations with C or C-E-G-treated macrophages. KPPC-ova cells were pre-stained with Lumiprobe^®^ DiR lipophilic tracer, a near-infrared fluorescent carbocyanine dye for cell membrane labelling prior to co-culture, and Annexin V NIR red dye was used to discern apoptotic cells. GFP fluorescence emanating from OT-NG CD8^+^ T cells indicates T cell activation. **d** Serial quantification of GFP positive (indicative of activated state) of OT-NG CD8^+^ T cells cultured alone or with the indicated cell types over time. **e** Number of GFP cells or (**f**) apoptotic KPPC-ova cells per field were measured using Incucyte imager over time to indicate the activation status of OT-NG CD8^+^ T cells and dead KPPC-ova cells, respectively. The experiment was done at least twice each in five biological replicates. *P* values calculated by two-way ANOVA followed by Tukey’s multiple comparison test

Although C-E-G may theoretically have cytotoxic effect on all CCR2-expressing cell types, scRNAseq analysis of autochthonous KPPC tumours showed that the dominant cell types diminished by C-E-G are TAMs and monocytes, whereas its effect on CAFs, dendritic cells and NKT cells are probably negligible due to low *Ccr2* expression (Suppl. Fig. S4a, S4b). Therefore, we focused on macrophages in subsequent in vitro experiments to study the impact and mechanism of action of C-E-G. We derived primary BMDMs from C57BL/6J mice, cultured them with conditioned media (CM) collected from KPPC cells and treated them with C, C-E, C-G or C-E-G at sublethal concentrations (4 mM) for two days (Fig. 4a). By FACS, C-G induced marked expression of M2-polarized markers ARG-1 and CD206, whereas C-E-G was less potent in inducing these markers. Additionally, C-E-G was more potent than C-G in inducing expression of M1 markers such as MHC I molecules H-2Kb, H2-Db and IFNg. Both C-G and C-E-G induced MHC II expression, although C-G is more potent than C-E-G (Fig. 4b). These results are consistent with scRNAseq data showing increased expression of these markers in C-E-G-treated TAMs (Suppl. Fig. S4c).

To test the function of C-E-G-treated TAMs, we developed a novel double transgenic OT-1;Nur77-GFP (OT-NG) mouse model wherein the CD8^+^ T cells express GFP driven by the Nur77 transcription factor upon activation of the OT-1 T cell receptor [14]. We co-cultured these OT-NG CD8^+^ T cells with KPPC cell expressing ovalbumin (KPPC-ova) and labelled with Nuclight Rapid NIR Dye^®^ (blue) dye, as well as BMDMs pretreated with C or -C-E-G for two days. Annexin V (red) NIR dye was added to the co-cultures which were then subject to live cell imaging using Incucyte^®^ to detect GFP as marker of T cell activation and red fluorescence to detect dead cells (Fig. 4c and d**)**. When cultured alone or with macrophages, OT-NG CD8^+^ T cells expressed very low level of GFP. However, when cultured with KPPC-ova and macrophages, we observed significantly higher and more sustained GFP expression especially with C-E-G treated macrophages (Fig. 4e), indicative of enhanced antigen presentation to OT-NG CD8^+^ T cells. Next, we measured the killing activity of OT-NG CD8^+^ T cells on KPPC-ova cells. It is worth noting that OT-1 CD8^+^ T cells alone are unable to eliminate ovalbumin-expressing tumour cells due to defective priming and antigen presentation [15]. Indeed, the killing effect of OT-NG CD8^+^ T cells was markedly augmented in the presence macrophages, and more so with C-E-G-treated macrophages (Fig. 4f). These results suggest that macrophages reprogrammed by C-E-G may exert anti-tumour effect through antigen presentation and activation of cytotoxic T cells.

### C-E-G enriches CCRL2-expressing TAMs that promotes CD8^+^ T cytotoxicity

**Fig. 5:**
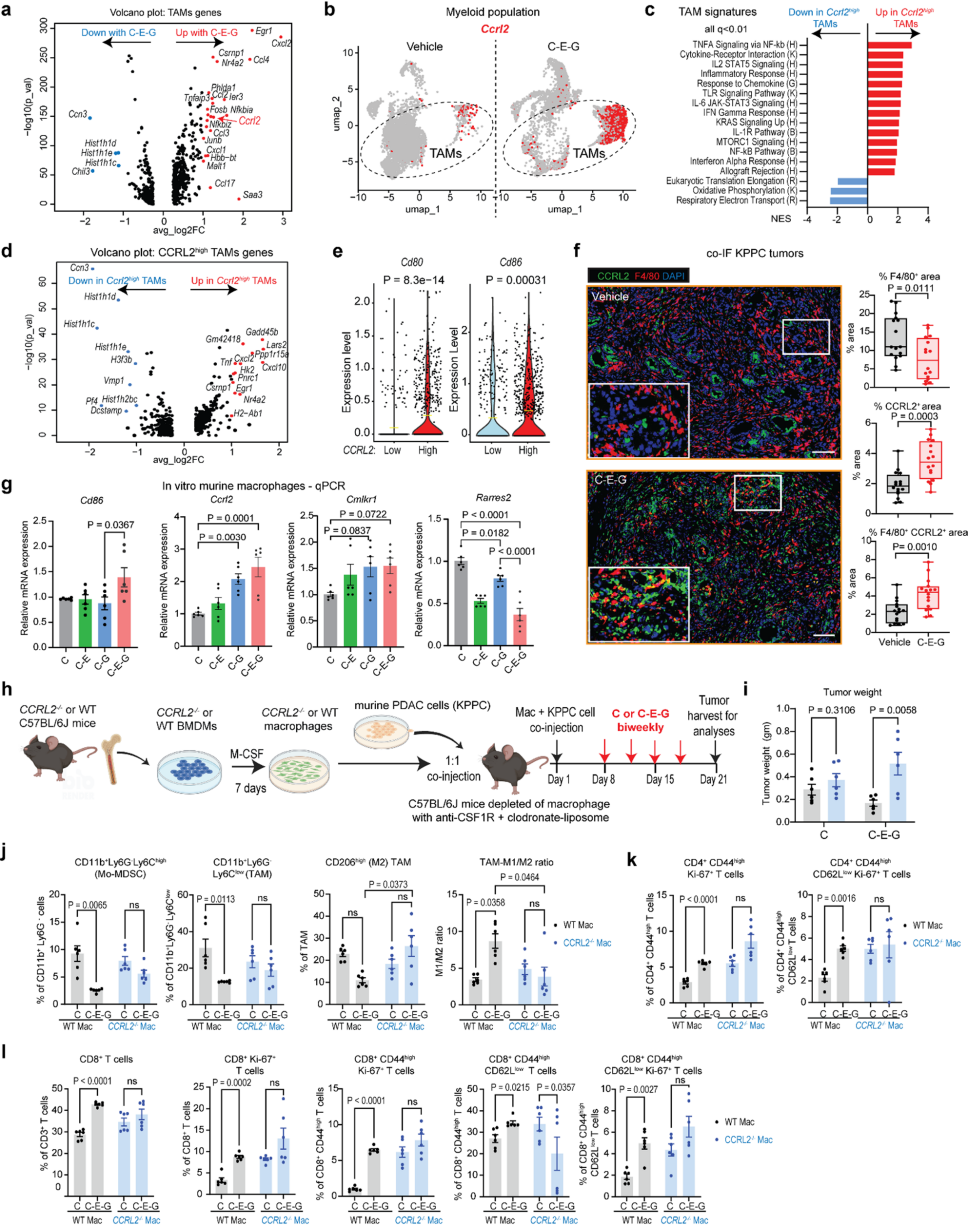
C-E-G enriches CCRL2-expressing TAMs that promotes CD8^+^ T cytotoxicity. **a** Volcano plot highlighting significantly upregulated and downregulated genes (> or < 2-fold change, *P* < 0.0001) between intratumoural TAMs from autochthonous KPPC mice treated with vehicle or C-E-G, based on scRNAseq in Fig. 3b. **b** UMAP plot showing increased abundance of CCRL2-expressing TAMs in autochthonous KPPC tumours treated with C-E-G. **c** Bar graph showing significantly up- or downregulated pathway signatures between Ccrl2high and Ccrl2low TAMs. **d** Volcano plot highlighting significantly upregulated and downregulated genes (> or < 2-fold change, *P* < 0.0001) between Ccrl2high and Ccrl2low TAMs. **e** Violin plots showing the increased expression of Cd80 and Cd86 in Ccrl2high TAMs. **f** Representative co-IF images and quantification of CCRL2, F4/80 and dual positive areas in vehicle or C-E-G-treated KPPC tumours (scale bars = 100 mm). **g** Quantitative RT-PCR showing relative expression of Ccrl2 and Cmklr1 genes in murine macrophages treated with the indicated nanotheranostics at 5 mM for 36 h. **h** Schematic illustrating co-transplantation of primary WT or Ccrl2−/− macrophages and KPPC cells orthotopically in syngeneic C57BL/6J mice pre-treated with anti-CSF1R and clodronate-liposome to deplete macrophages. One week after transplantation, mice were treated with C or C-E-G biweekly for two weeks and the tumours were harvested 4 days after the last dose for analyses. **i** Bar graph showing the tumour weights of the indicated transplanted KPPC tumours treated with C or C-E-G. FACS-based quantification of the indicated intratumoural (**j**) myeloid, (**k**) CD4+ T cells and (**l**) CD8+ T cells from the indicated transplanted KPPC tumours

Next, we sought to determine the mechanisms underlying the T cell-promoting function of C-E-G-programmed TAMs. Our scRNAseq data showed that C-E-G-treated TAMs were enriched for expression of inflammatory chemokines including *Ccl17*,* Ccl2*,* Ccl3*,* Ccl4*,* Cxcl1* and *Cxcl2*. It is well-documented that small molecule CCR2 inhibitors such as BMS-813,160 and PF-0413630 which reversibly block CCR2 receptors on monocytes, rather than killing them [5, 10, 16], result in compensatory upregulation of circulating CCL2 levels, which are likely produced by CCR2^low^ monocytes/TAMs to mobilize more monocytes [6]. Interestingly, C-E-G-treated TAMs also expressed higher level of chemokine (C-C motif) receptor-like 2 (*Ccrl2*, termed CCRL2^high^ TAMs, Fig. 5a). CCRL2 is a seven-transmembrane domain receptor that is known to bind and concentrate chemerin, a chemoattractant for CD8^+^ T cells, NK, and other innate cells [17]. Analysis of the GEPIA2 databased showed that higher expression of *CCRL2* trended towards a better overall survival for patients with basal subtypes of PDAC, as well as a weak positive (*R* = 0.2) correlation between *CCRL2* and *CCR2* expressions (Suppl. Fig. S5a, 5b). 

Interestingly, UMAP analysis of scRNAseq data showed *Ccrl2*^high^ TAMs to be transcriptionally distinct from the *CCR2*^+^ TAM population that was diminished with C-E-G (Fig. 5b). Compared to *Ccrl2*^low^ TAMs, *Ccrl2*^high^ TAMs displayed upregulated inflammatory signatures including TNFA, IL-1, IL-6, IL-2 and IFNg pathways (Fig. 5c), and expression of *Tnf*,* Cxcl2*,* Cxcl10*,* Egr1 and Csmp1* that were also seen in C-E-G-treated TAMs (Fig. 5d). Upregulation *Ccl2* expression was not seen in *Ccrl2*^high^ TAMs. Notably, *Ccrl2*^high^ TAMs have higher expression of co-stimulatory genes *Cd80* and *Cd86* (Fig. 5e), consistent with the known role of CCRL2 in drawing cytotoxic T cells. In parallel, multiplex IF of C-E-G-treated PDAC showed decreased F4/80^+^ area, as well as increased CCRL2^+^ and dual F4/80^+^ CCRL2^+^ areas, confirming an increased abundance of CCRL2-expressing TAMs (Fig. 5f). In cultured primary murine macrophages, C-E-G treatment induced higher expression of *Cd86* and *Ccrl2*, but not *Cmlkr1* which encodes chemerin chemokine-like receptor 1, another receptor that binds *Rarres2* which encodes chemerin (Fig. 5g). These data led us to hypothesize that enrichment of *Ccrl2*^high^ TAMs underlies the mechanism of action for C-E-G. To confirm this, we derived BMDMs from WT or *Ccrl2*-knockout (*Ccrl2*^*−/−*^) C57BL/6J mice [18], and co-cultured them with freshly isolated splenic T cells from C57BL/6J mice in the presence of CM from KPPC cells for two days (Suppl. Fig. S5c). FACS analysis revealed that BMDMs from WT, but not from *Ccrl2*^*−/−*^ mice, induced higher expression of IFNγ and CD107 indicative of cytotoxicity [19] on T cells (Suppl. Fig. S5d). Compared to WT, *Ccrl2*^*−/−*^ macrophages exhibited lower expression of the antigen-presenting molecule MHC II and higher levels of the chemokine CCL2 (Suppl. Fig. S5e). In conjunction with our scRNAseq data, we deduce that CCL2 is probably produced by *Ccr2*^*low*^ *Ccrl2*^low^ TAMs as an escape mechanism to C-E-G.

To study the function of CCRL2 in TAMs, we performed co-culture experiments using OT-NG CD8⁺ T cells, KPPC-OVA cells, and either WT or *Ccrl2*^*−/−*^ macrophages (Suppl. Fig. S5f). Interestingly, OT-NG CD8⁺ T cells co-cultured with *Ccrl2*^*−/−*^ macrophages initially expressed higher levels of GFP within the first 12 h compared to those co-cultured with WT macrophages; however, this activation was not sustained over time (Suppl. Fig. S5g). Moreover, OT-NG CD8⁺ T cells co-cultured with WT macrophages exhibited more robust cytocidal activity against KPPC-OVA cells than those cultured with *Ccrl2*^*−/−*^ macrophages (Suppl. Fig. S5h). These findings strongly suggest a critical role for CCRL2-expressing macrophages in promoting effective cytotoxic T cell response.

### CCRL2-expressing TAMs supports anti-tumour T cell immunity in vivo

To confirm the role of CCRL2 in anti-tumour T cell immunity in vivo, we performed macrophage transplant experiment in mice. We isolated BMDMs from WT or *Ccrl2*^*−/−*^ mice, differentiated them into primary macrophages and co-injected them 1:1 with KPPC cells orthotopically into the pancreas of C57BL/6J mice which had been depleted of macrophages with anti-CSF1R and clodronate liposomes, as described [20]. One week later the mice were treated with C or C-E-G biweekly for two weeks, followed by euthanasia and tumour harvest 4 days after the last dose, as done in previous experiments (Fig. 5h). While both WT and *Ccrl2*^*−/−*^ macrophages, produced similar tumour weight in mice treated with C, only tumours co-transplanted with WT, but not *Ccrl2*^*−/−*^ macrophages, showed a significant reduction in size following C-E-G treatment (Fig. 5i). FACS analysis showed that the inhibitory effects of C-E-G on monocytic MDSCs, TAMs, as well as M1 polarization were abrogated in tumours transplanted with *Ccrl2*^*−/−*^ macrophages (Fig. 5j, Suppl. Fig. S5h). Additionally, while in this model C-E-G treatment increased proliferating CD4^+^ effector, CD4^+^ effector memory T cells, total and proliferating CD8^+^ T cells, proliferating effector CD8^+^ T cells, total and proliferating effector memory CD8^+^ T cells in control tumours, these effects were abrogated in tumours transplanted with *Ccrl2*^*−/−*^ macrophages (Fig. 5k and l, Suppl. Fig. S5i). Together, these data demonstrate that CCRL2^+^TAMs are required for C-E-G-induced T cell immunity.

To determine the origin of the CCRL2⁺ TAMs, we leveraged Tim4 as a marker to distinguish tissue-resident TAMs (Tim4⁺) from monocyte-derived TAMs (Tim4⁻) [21]. While C-E-G treatment led to a significant increase the abundance of CCRL2⁺ TAMs in orthotopic KPPC tumours, no difference between Tim4⁺ and Tim4⁻ subsets was seen in vehicle or C-E-G treated tumours (Suppl. Fig. S5k), suggesting both pre-existing tissue-resident and monocyte-derived TAMs contributes to CCRL2^high^ TAMs. While KPPC tumours inoculated in *Ccr2* knockout *(CCR2*^*gfp/gfp*^*)* mice [22] were smaller compared to those grown in WT mice, supporting the pro-tumourigenic role of CCR2^+^ TAMs, their TAMs still expressed CCRL2. While C-E-G treatment resulted in smaller KPPC tumours with more CCRL2⁺ TAMs in WT mice, it had no impact on KPPC tumours grown in *Ccr2* knockout mice (Suppl. Fig. S5i). These results suggest that the CCRL2^high^ TAMs induced by C-E-G were derived from both monocyte-derived and tissue-resident populations that have low or absent CCR2 expression and are therefore spared by C-E-G.

### CCRL2⁺ TAMs concentrate intratumoural chemerin to recruit and activate T cell immunity

**Fig. 6:**
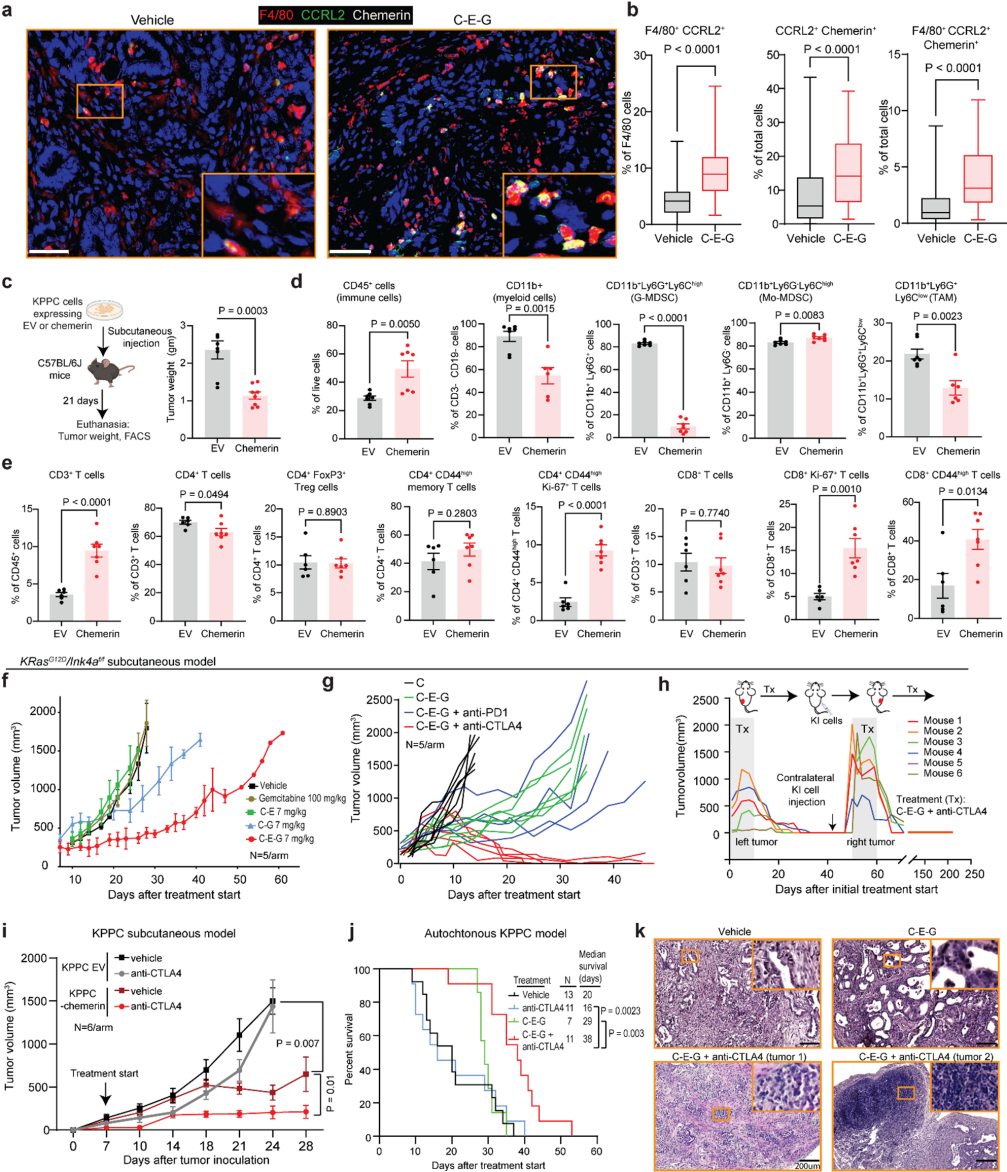
CCRL2⁺ TAMs concentrate intratumoural chemerin to recruit and activate T cell immunity. **a** Representative IF images and (**b**) Quantification of the abundance of double F4/80+ CCRL2+, CCRL2+ Chemerin+ and triple F4/80+ CCRL2+ Chemerin+ cells in vehicle or C-E-G-treated KPPC tumours. 4–5 tumours/arm and 8–10 fields/tumour covering the entirety of each tumour were analyzed (scale bars = 100 mm). (**c**) Experimental schematics of subcutaneous implantation of EV or chemerin-overexpressing KPPC cells in C57BL/6J mice and the final tumour weight at euthanasia. FACS-based quantification of the indicated intratumoural (**d**) Myeloid and (**e**) T cells from EV or chemerin-overexpressing KPPC tumours (*N* = 6/arm). *P* values were calculated by two-tailed unpaired t test or two-way ANOVA followed by Tukey’s multiple comparison test. (**f**, **g**) Tumour growth curves of subcutaneous KI tumours in FVBN/J mice treated as indicated when tumours grew to ~ 100 mm3 in volume (*N* = 5/arm in each experiment). **h** Tumour growth curves of subcutaneous KI tumours treated with biweekly C-E-G plus anti-CTLA4 when tumours grew to ~ 50–100 mm3 in volume until complete regression. Treated mice were rechallenged with KI cell implantation at the contralateral flank, observed and re-treated with biweekly C-E-G plus anti-CTLA4. **i** Tumour growth curves of subcutaneous KPPC tumours stably expressing EV or chemerin treated with either vehicle or anti-CTLA4 biweekly when tumours grew to 50–100 mm3 in volume (*N* = 6/arm). **j** Kaplan-Meier survival analysis of autochthonous KPPC mice treated as indicated starting from 6 weeks of age till humane endpoint. **k** Histology of end-stage KPPC tumours treated as indicated and harvested when mice reached humane endpoints (scale bar = 200 mm)

The only known ligand for CCRL2 is chemerin [23], a chemoattractant for CD8^+^ T cells. Enforced expression of chemerin in tumour cells is sufficient to recruit CD8^+^ T cells and suppress tumour growth in preclinical melanoma, breast and prostate cancer models [19, 24, 25]. Therefore, we hypothesize that CCRL2 macrophages increase intratumoural chemerin to attract CD8^+^ T cells. Supporting this notion, by multiplex IHC C-E-G treated KPPC tumour displayed higher abundance of F4/80^+^ CCRL2^+^ cells that also co-stained with chemerin (Fig. 6a and b). To discern the role of chemerin alone in recruiting CD8^+^ T cells, we stably overexpressed chemerin or an empty vector (EV) in KPPC cells and perform subcutaneous transplantation in C57B/6J mice. Compared to EV tumours, chemerin-expressing tumours were smaller (Fig. 6c), had more CD45^+^ immune cells, less myeloid cells, granulocytic MDSCs and TAMs (Fig. 6d), and were infiltrated with more T cells, proliferating CD4^+^ memory T cells, proliferating CD8^+^ T cells and CD8^+^ memory T cells (Fig. 6e). In another subcutaneous experiment, chemerin overexpressing tumours were smaller compared to control tumours and similar to C-E-G-treated control tumours. Yet, C-E-G still resulted in further mild decrease of the weight of chemerin-expressing tumours (Suppl. Fig. S6a). These results showed C-E-G enriches for CCRL2 TAM which concentrates intratumoural chemerin as the main mechanism to draw and activate cytotoxic T cells.

Although C-E-G treatment alone reduced tumour weight in autochthonous KPPC mice, it had minimal direct impact on neoplastic cells (Fig. 2c and d), highlighting the need for combination strategies that can eradicate PDAC cells. The immunomodulatory effects of C-E-G, particularly its ability to suppress TAMs and enhance the cytotoxicity of antigen-specific OT-NG CD8^+^ T cells, led us to explore its combination with ICB. To test this, we utilized the KI xenograft mouse model, in which tumours can grow subcutaneously to approximately 2000 mm³, enabling us to assess and compare the efficacy of various ICB combinations. As with other PDAC models, ^64^Cu-C-E-G accumulated robustly within subcutaneous KI tumours (Suppl. Fig. S6b). As a single agent, C-E-G was significantly more effective at suppressing tumour growth compared to C-G or gemcitabine administered at a 13-fold higher concentration (Fig. 6f). At euthanasia, vehicle, and C-E-G -treated mice showed no difference in hemogram, blood chemistries that include liver and kidney functions, body or organ weight (Suppl. Fig. S6c-e). We previously published that 1 h after intravenous injection, C-E-G is detected in multiple organs and is largely cleared after 24 h, suggesting that it can cross the endothelial barrier in normal organs and tumour tissues [8].

Next, we study the impact or C-E-G on monocytes in peripheral blood and bone marrow to better understand its systemic toxicities. C57BL/6J mice bearing orthotopic KPPC tumours were treated with vehicle, or C-E-G for two weeks (Suppl. Fig. S6f). In this model, monocytes are present in the tumour, bone marrow or blood, but < 10% of these monocytes expressed CCR2 by FACs at baseline and these did not significantly decrease with C-E-G (Suppl. Fig. S6g). In the scRNAseq of autochthonous KPPC tumour, ~ 20% of intratumoural TAMs and ~ 10% of intratumoural monocytes express *Ccr2* (Fig. 3b and c), which is consistent with published data on human PDAC patients showing less than 15% of PBMC and only ~ 10% of bone marrow mononuclear cells are CCR2+ [10]. The low fraction of CCR2 + monocytes probably explained why C-E-G has not measurable impact on total monocytes population in these locations. However, C-E-G did result in mild statistically significant decrease of intratumoural CCR2^+^ monocytes (Suppl. Fig. S6h), increased apoptotic monocytes in peripheral blood and tumour(Suppl. Fig. S6i), but no significant change of CCR2 + EpCAM+, CAFs, CD4 or CD8 T cells (Suppl. Fig S6j). The lack of effect of C-E-G on these cells could be explained by low *Ccr2* expression in tumour cells, CAFs and T cells, as supported by scRNAseq data (Suppl. Fig. S4b), and/or higher resistance of these cells to gemcitabine (Suppl. Fig. S2e).

From the scRNAseq data of C-E-G-treated KPPC tumours, we found CTLA4 as the most upregulated checkpoint molecule (Suppl. Fig. S6k), suggesting it as a key exhaustion mechanism. We next tested C-E-G in combination with anti-CTLA4, or anti-PD1 since this is another commonly used ICB in the clinic, in the subcutaneous KI model. We found that the combination of C-E-G plus anti-CTLA4, but not anti-PD1, led to complete tumour regression (Fig. 6g). To determine whether the combination of C-E-G plus anti-CTLA4 can induce anti-tumour memory, we treated with tumour bearing FVBN/J mice with C-E-G plus anti-CTLA4 until their tumours had completely regressed for at least 10 days. KI cells were then injected into the contralateral flanks of these mice. However, the injected KI tumours still grew at a similar rate as during the initial injection, indicating that the treatment do not raise T memory cells. Strikingly, when re-treated with C-E-G and anti-CTLA4, we observed complete regression of tumours as large as ~ 2000 mm³ (Fig. 6h), attesting to the robust anti-tumour efficacy of this combination. Because C-E-G functions through enrichment of CCRL2^+^ TAMs and chemerin, we tested whether chemerin overexpression alone may render PDAC cells responsive to anti-CTLA4. Indeed, KPPC tumours stably expressing chemerin not only had slower growth kinetics when xenografted in C57BL/6J mice, they responded to the suppressive effect of anti-CTLA4 alone (Fig. 6i), supporting chemerin enrichment as the mechanism of action of C-E-G. Finally, we tested C-E-G plus anti-CTLA4 in autochthonous KPPC mice, a challenging model due to the aggressive and highly desmoplastic nature of these tumours. Consistent with the KI model, the combination of C-E-G plus anti-CTLA4 significantly prolonged the survival of KPPC mice (Fig. 6j). Histologic analysis of combo-treated end-stage tumours revealed extensive areas of necrosis, dense lymphocyte infiltration, and the presence of tertiary lymphoid structures (Fig. 6k), a hallmark of a robust anti-tumour immunity. Together, these findings strongly support advancing this therapeutic combination into clinical trials for PDAC patients.

## Discussion

Here, we describe the development of a CCR2-targeted nanotheranostic, C-E-G, designed to selectively deliver a cytotoxic payload to CCR2⁺ TAMs, and its preclinical efficacy across different PDAC models. The fundamental mechanism of action of C-E-G is targeted killing of CCR2⁺ TAMs with gemcitabine, akin to antibody–drug conjugates such as trastuzumab deruxtecan (Enhertu), capitalizing on the high expression of CCR2 in TAMs and their greater susceptibility to gemcitabine compared with other cell types. As such, C-E-G depletes immunosuppressive CCR2⁺ TAMs and results in enrichment of CCRL2⁺ macrophages from the remaining tissue-resident and monocyte-derived TAMs, which recruit and activate T cells via chemerin.

Our transcriptomic and scRNA-seq analyses indicate that CCRL2⁺ TAMs represent a distinct subset with the potential to bridge innate and adaptive immunity. This is supported by macrophage transfer experiments showing that *Ccrl2*-deficient macrophages fail to activate CD8⁺ T cells in response to C-E-G both in vitro and in vivo. Chemerin, the canonical ligand for CCRL2, was upregulated within the TME following C-E-G treatment. Prior studies have shown that chemerin promotes T and NK cell trafficking via binding to CMKLR1, a receptor expressed on effector lymphocytes [19]. Interestingly, C-E-G did not induce CMKLR1 expression in TAMs, indicating that chemerin’s immunostimulatory effects in this setting are unlikely to be mediated through myeloid CMKLR1 signaling. Instead, we speculate that chemerin produced by CCRL2⁺ TAMs enhances recruitment and activation of CMKLR1⁺ effector memory CD8⁺ T cells. Further investigation into the dynamics and cellular sources of chemerin in the TME will be important to optimize therapeutic use of this axis.

Besides killing TAMs inside the tumour, C-E-G may engage and trigger the apoptotic program of monocytes in the peripheral blood, which may proceed to full cell death upon entering the TME. It is well established that apoptotic cells can induce immune tolerance by producing reactive oxygen species which oxidize HMGB1 and blunt immunogenicity [26, 27]. This may also partly explain why C-E-G as monotherapy suppress tumour growth but did not result in tumour regression. Therefore, besides combining with ICB, therapeutic strategies that enhance clearance of these apoptotic debris in the TME may augment the efficacy of C-E-G.

Our study has the following limitations. First, although we employed multiple state-of-the-art including the KPPC mouse models, they do not develop spontaneous metastases and thus may not fully capture human myeloid heterogeneity. Second, not all immunosuppressive macrophages in PDAC express CCR2, as shown by our analysis using human PDAC TMA and scRNAseq in KPPC tumours. Therefore, CCR2^low^ immunosuppressive macrophage populations may evade C-E-G-mediated depletion by upregulating other chemokines or cytokines such as *Cxcl2*,* Cxcl10*,* Il1b*,* Tnf*, and *Vegfa* as our data showed. Upregulation of *CXCL2* attracts CXCR2⁺ neutrophils as a mechanism of resistance to CCR2 inhibition [28] and thus could be a resistance mechanism to C-E-G. Additionally, upregulation of *Il1b*,* Tnf*, and *Cxcl10*, all of which have been implicated in driving inflammatory TAM phenotypes. Previous studies have shown that TAMs expressing IL-1β, TNF, and VEGFA exhibit an inflammatory phenotype that promotes PDAC progression and is associated with poor survival [29, 30]. Therefore, TAMs are a heterogeneous population capable of rapidly altering gene expression and phenotype under therapeutic stress. Although C-E-G showed no overt toxicity in mice, formal toxicology and pharmacokinetic studies in large animals will be required for translation. In future clinical trials, companion diagnostics to assess tumour CCR2 expression are needed to evaluate on-target effects and predict patient response as part of personalized medicine.

In summary, our study provides compelling evidence that a CCR2-targeted cytotoxic nanotheranostic agent not only depletes CCR2⁺ TAMs but also enriches CCRL2⁺ TAMs that recruit and activate cytotoxic T cells, thereby supporting its advancement into clinical trials for PDAC patients.

## Materials and methods (Please see supplementary data for more details)

### Study approval

The study was approved by the Washington University Institutional Animal Care and Use Committee (IACUC) under Protocols #22–0101 and #22–0260.

### Mouse strains

KPPC (p48-Cre; *Trp53*^*Flox/Flox*^; LSL-*Kras*^*G12D*^) PDAC mouse breeding pairs were originally from Jackson Laboratories and were maintained and genotypes as published [11]. The Nur77-GFP (C57BL/6-Tg(Nr4a1-EGFP/cre)820Khog/J, strain 016617) and OT-1 (C57BL/6-Tg (TcraTcrb)1100Mjb/J, strain 003831) mice were purchased from Jackson Laboratories, crossed, and genotyped as recommended to generate double transgenic OT-NG mice. *Ccrl2* knockout mice were published [31], kindly provided by Dr. Brian Zabel, bred in-house and genotyped as recommended. *Ccr2*^*gfp/gfp*^ mice [22]were provided by Dr. Kory Lavine.

### Human PDAC tissue microarray (TMA)

PDAC TMA were obtained from Department of Surgery at Washington University with IRB approved (#201702130). All human tissue studies were performed by ethical principles of the Declaration of Helsinki. Automated staining of TMA slides was conducted using antibodies to CD8, CD163, CD11b, CCR2, and CK19 (see Supplementary Table 1) on the BOND RXm system (Leica Biosystems), with antigen retrieval and detection using Bond Polymer Refine Detection (DS9800). Slides were dehydrated, mounted, and scanned with a Zeiss Axio Scan Z1 Slide Scanner using a 20× objective for whole-slide digital imaging. Data was analyzed using HALO software (Indica Labs) with Area Quantification, Cytonuclear, or HighPlex modules.

### In vivo xenograft experiments

For orthotopic and subcutaneous PDAC models, 8–12 weeks old C57BL/6J or FVB/NJ mice were used for KPPC or KI cell lines, respectively. For subcutaneous implantation, 40,000 to 50,000 cells suspended in 100 µL of MatriGel: PBS (1:1) mixture were injected into the flank of mice. Tumour volumes were measured twice weekly using calipers and calculated using the formula: volume = (length × width²)/2. For orthotopic models, tumours were surgically implanted into the pancreas with 20,000 to 40,000 cells in 50 µL of Matrigel: PBS mixture using a 30-gauge insulin syringe. Orthotopic tumour volumes were assessed following isoflurane sedation through manual palpation. Following implantation, mice were randomized into treatment groups based on tumour volume or gross palpation. At humane or defined experimental endpoints, mice were euthanized using carbon dioxide followed by cervical dislocation. All experiments were done in compliance with Washington University’s Institutional Animal Studies Committee guidelines under approval (#2022 − 0161).

### Quantification and statistical analysis

Statistical analyses were performed using Prism software (GraphPad), with input from the Biostatistics Core at Washington University. Data represents at least two independent experiments unless stated otherwise. For tumour experiments, animals were randomized by a blinded investigator based on ascending tumour sizes. Statistical comparisons between two groups were made using unpaired Student’s two-tailed t-tests. For multiple groups, one-way or two-way ANOVA with the appropriate post-tests was applied. Data are presented as mean ± SEM, with *P* < 0.05 considered significant. Cox proportional hazards models were used for survival analyses, and log-rank (Mantel-Cox) test was used for survival comparisons.

## Supplementary Information

Supplementary Material 1.

## Data Availability

All data is available in the main text or the supplementary materials and can be obtained by contacting the corresponding authors. The RNA-seq data generated in this study have been deposited in the Gene Expression Omnibus (GEO) under accession number GSE296754.
