# Supplementary material for: Therapeutic reprogramming of tumour-associated macrophages in pancreatic cancer using a cytotoxic CCR2-targeted nanotheranostic
Source: Mol Cancer. 2026 Feb 7;25:65. doi: 10.1186/s12943-026-02597-7 (PMC12977780; doi:10.1186/s12943-026-02597-7)
Supplement: Supplementary file 1 — Supplementary Material 1. [file 12943_2026_2597_MOESM1_ESM.docx]

**Therapeutic Reprogramming Of Tumour-Associated Macrophages In Pancreatic Cancer Using A Cytotoxic CCR2-Targeted Nanotheranostic**

Vikas Kumar Somani^1, §^, Xiaohui Zhang^2, §^, Timothy Hung-Po Chen^1^, Ashenafi Bulle^1^, Sapana Bansod^1^, Lin Li^1^, Yutong Geng^1^, Liang-I Kang^1^, Gyu Seong Heo^2^, Hannah Luehmann^2^, Yuena Zhang^2^, Muhammad A. Saeed^1^, Kory J. Lavine^3^, David G. DeNardo^1^, Russell K. Pachynski^1^, Yongjian Liu^2, *^, Kian-Huat Lim^1, *^

^1^Division of Oncology, Department of Internal Medicine, Washington University School of Medicine, St. Louis, Missouri, United States

^2^Department of Radiology, Washington University School of Medicine, St. Louis, Missouri, United States

^3^Division of Cardiology, Department of Internal Medicine, Washington University School of Medicine, St. Louis, Missouri, United States.

^§^ These authors contributed equally; ^*^shared senior authorship

The authors declare that they have no competing interests.

**Supplementary Material and Methods, Figures and Figure Legends**

*Corresponding author:

Kian-Huat Lim, MD PhD

Washington University School of Medicine

660 South Euclid Avenue, Campus Box 8069

Saint Louis, MO 63110

Tel: 314-362-6157, Fax: 314-747-9329

Email: [kian-huat.lim@wustl.edu](mailto:kian-huat.lim@wustl.edu)

**MATERIALS and METHODS**

**Study rigors**

To ensure scientific rigor, all animal experiments including treatment and tumour measurements and subsequent analyses were performed in a blinded manner by independent team members. In vitro experiments were replicated two to four times to confirm reproducibility.

**Synthesis of copper nanoclusters (CuNC):**

The synthesis of CEG, CE, CG, and C were following our previous method ^1,2^. Typically, for imaging studies, CuCl_2_ (376 µL, 10 mM), TA-PEG ligands (400 µL, 2.5 mM (CEG used 1:2:0 molar ratio of TA-PEG-ECL1i, TA-PEG-Gem and TA-PEG-OMe; CE used 1:0:2 molar ratio of TA-PEG-ECL1i, TA-PEG-Gem and TA-PEG-OMe; CE used 0:2:1 molar ratio of TA-PEG-ECL1i, TA-PEG-Gem and TA-PEG-OMe; and C used all TA-PEG-OMe), and ^64^CuCl_2_ (0.1 M NH_4_OAc, pH 5.5, *ca*. 18.5 MBq µL^-1^) were mixed in 2 mL water and stirred for 15 mins at RT. Then, sodium borohydride (450 µL, 20 mM) was added to the reaction vial with rapid stirring for another 25 mins. The prepared nanoparticles were then immediately centrifuged with a centrifugal filter unit (Amicon Ultra, 10 kDa NMWL, 7500 g, 15 mins) and washed 3 times with water. The final product was diluted with sterile water and immediately delivered for PET imaging. Radiochemical purity was measured by instant radio-thin layer chromatography (iTLC or radio-TLC) using glass microfiber chromatography paper impregnated with a silica gel (Agilent Technology) and 10% ammonium acetate and methanol (1:1 volume ratio) mixture as developing solution (Radio-TLC, BioScan). For treatment study, the synthesis of nanoclusters was scaled up according to mouse numbers using the same method without adding ^64^CuCl_2_. The prepared nanoclusters were filtered through 0.22 μm filter (Corning® 50 mL Tube Top Vacuum Filter System) prior to centrifugation, which was injected through IV immediately after purification. For in vivo experiments, C, C-E, C-G, C-E-G were prepared in complete media at 5μM and given intravenously (7–10 mg/kg, twice weekly). Treatment began when tumours reached 100–150 mm³ and continued until tumours exceeded 2000 mm³ or humane endpoints were met. For immune checkpoint blockade, mice received anti-CTLA4 (UC10-4F10-11, BioXcell), anti-PD1 (RMP1-14, BioXCell) by IP injection twice per week at 250 µg and 200 µg, respectively.

**Cell lines and plasmids**

The KI cell line was provided by Dr. David DeNardo. The KPPC cell lines were derived from the PDAC of autochthonous KPPC mice at 8 weeks of age. KPPC-ova cells were generated via lentiviral expression of ovalbumin. KPPC-chemerin overexposing cell line was generated using pCMV3-RARRES2 plasmid provided by Dr. Russell Pachynski, and the resultant cells were selected using hygromycin. 293T cells were purchased from ATCC (cat. #CRL-3216). All cell lines were cultured in DMEM supplemented with 10% fetal bovine serum and 1% penicillin/streptomycin. Mycoplasma testing was performed every 6 months using MycoSEQ Detection kit (Applied Biosystems). All lines were used for fewer than 4 months after revival from cryopreservation.

**Viability assay**

KPPC cells were seeded in 24-well plates (40-50% confluency) and treated with 5 µM C, C-E, C-G, or C-E-G. Cell viability was monitored using the Incucyte S3 system, capturing phase contrast images every 4 hours for 24 hours. Confluency and density were quantified with Incucyte software, and data were normalized. Nine images per time point were analyzed per well to calculate mean values.

**Cell-drug adherence analysis**

KPPC cells were incubated in DMEM at 37°C with 5% CO₂ for adherence. C, C-E, C-G, or C-E-G conjugated with Texas Red (TR) were added (5 µM). After 6 hours, cells were washed, stained with DAPI, and fixed with 10% paraformaldehyde. Imaging was performed using an Axio Z2 fluorescent microscope.

**Isolation of BMDMs and splenic T cells**

Bone marrow-derived macrophages (BMDMs) were isolated from WT and *Ccrl2* KO mice. Femurs and tibiae were collected, disinfected, and bone marrow flushed using PBS. Cells were seeded in BMDM medium with M-CSF and cultured at 37°C for differentiation. Mature BMDMs were obtained in 6–7 days. Differentiated BMDMs were harvested, counted, and seeded in 96-well plates with tumour conditioned media (CM). Media was refreshed with M-CSF, and cells were stimulated with TCM for RNA isolation or other experiments.

Splenic T cells were isolated from C57BL/6J mice using magnetic beads (Miltenyi cat. #130-095-236), enriched, and plated in anti-CD3ε-coated wells with CD28 for overnight proliferation. Depending on experiments, BMDMs were primed with CM and treated with C, C-E, C-G, C-E-G for 48 hours. After these procedures, BMDMs were washed, and T cells were added for co-culture.

**Multiplex IF of murine PDAC**

For mouse PDACs, embedded tissues were sectioned into 6 μm sections and stained using antibodies to CCRL2, CCR2, CD11b, F4/80, CD8, neutrophil elastase, and CK19 (see **Supplementary Table 1**). Staining was performed using the BOND RXm system, with antigen retrieval, primary antibody incubation, and chromogenic detection using AEC substrate. After each staining cycle, slides were manually stained with haematoxylin, scanned with Axio Scan.Z1, and destained before the next cycle. Citrate-based antigen retrieval was applied before each staining cycle.

**Analysis of multiplex IF data**

Images from different staining cycles were cropped into segments, deconvoluted using Deconvolution (Version 1.0.4; Indica Labs), and fused using HALO® (Indica Lab) software. Markers were pseudo-coloured and quantified using High Plex FL software in HALO. Ten 200x or 400x fields per tumour were analyzed by two individuals (K.H. Lim and M. Ruzinova), and data were presented as mean ± SEM. H&E sections were interpreted independently, and representative data were agreed upon by all members.

**Flow Cytometry**

After harvesting and digesting tumour tissues, single-cell suspensions were resuspended in flow cytometry (FC) buffer (PBS containing 1% BSA), and Fc receptors were blocked with rat α-mouse CD16/CD32 antibodies (Ebioscience, Santa Clara, CA, USA) for 10 minutes, followed by centrifugation. The cells were then labeled with 100 μL of fluorophore-conjugated α-mouse extracellular antibodies at the recommended dilutions for 25 minutes on ice. After labeling, the cells were fixed and washed with FC buffer. Intracellular staining was performed using the EBioscience buffer set. For cell fixation and permeabilization, BD Cytofix/Cytoperm™ Fixation and Permeabilization Solution (Catalog #554722) and BD Perm/Wash™ Buffer (Catalog #554723) were used as per the manufacturer’s instructions. Alternatively, the BD Cytofix/Cytoperm™ Fixation/Permeabilization Kit (Catalog #554714) was used for more efficient intracellular staining preparation. For apoptosis analysis (Annexin V/PI staining), Cells were stained with Annexin V-FITC and propidium iodide (PI) (BD Biosciences) and acquired by flow cytometry (FACS Calibur). Apoptotic cells (early and late) were quantified using FlowJo (v 10.10.0 software). Data was analyzed and graphed with GraphPad Prism (v7/8). All antibodies are listed in the key resources table (**Supplementary Table 1**). Data was acquired on a BDX-20 (BD Biosciences) and analyzed using FlowJo software (v10.10.0).

**Quantitative Real-Time PCR (qRT-PCR)**

Total RNA was extracted using RNAzol RT (Sigma). Complementary DNA (cDNA) was synthesized using the High-Capacity cDNA Reverse Transcription Kit (Thermo Fisher). Quantitative real-time PCR (qRT-PCR) was performed on an ABI 7900HT system using SYBR Green reagent (Applied Biosystems) and TaqMan primer-probe sets (Applied Biosystems). Gene expression was calculated using the comparative Ct (ΔΔCt) method and normalized to reference genes including GAPDH, Annexin, or hypoxanthine phosphoribosyltransferase (HPRT). Primer sequences are listed in Extended Data Table. Results are presented as mean ± SEM from biological replicates (n = 2–3).

**Mouse studies**

*Ccr2^gfp/gfp^* (JAX lab, 027619) mice were used to genetically label and ablate CCR2-expressing monocytes/TAMs in mechanistic studies.

**Animal models and tumour implantation:**

Eight-week-old C57BL/6J wild-type mice and *Ccr2^gfp/gfp^* mice were used for in vivo studies. Tumours were established using either KPPC wild-type or KPPC CCR2-deficient (sg*Ccr2*) pancreatic cancer cell lines. Cells were implanted either subcutaneously or orthotopically into the pancreas, as indicated. For orthotopic studies, mice were randomized 7 days post-implantation to receive vehicle, C-E, C-G, or C-E-G treatment and were monitored for 14 days (n = 6 per group). Treatments were administered intravenously twice weekly at the indicated doses. At study endpoint, tumours, blood, and bone marrow were harvested for downstream analyses.

**Micro-PET/CT imaging and autoradiography of ex vivo tumours**

Tumour-bearing mice were anesthetized and injected with 3.7 MBq ^64^Cu-CEG, ^64^Cu-C, or ^64^Cu-CEG blocking with 500 times cold CEG in 100 μL saline *via* the tail vein. Small animal PET scans were carried out on Inveon PET/CT system (Siemens, Malvern, PA) at 24 h post injection (60 min frame). The attenuation, scatter, normalization, and camera dead time were all corrected for micro-PET images for co-registration with micro-CT images. The Inveon PET/CT scanner is periodically calibrated using a normalization phantom of known activity concentration to ensure its quantitative accuracy. Micro-PET images were reconstructed with the maximum a *posteriori* (MAP) algorithm. Data quantification was carried out using Inveon Research Workplace. Tumour accumulation was computed as percent injected dose per gram (%ID/gram) of tissue in three-dimensional region-of-interests without the correction for partial volume effect. For autoradiography, the mice were perfused with saline transcardially, and the tumours were collected and sliced immediately following PET/CT scan. The slices were covered by a phosphor-imaging film plate and exposed overnight. The film was then imaged with a GE Typhoon FLA 9500 Biomolecular Imager.

**Mouse toxicity studies**

At euthanasia, KI-bearing mice treated with vehicle of C-E-G were submitted to Division of Comparative Medicine (DCM) research animal diagnostic laboratory in Washington University in St. Louis for clinical pathologic and histopathologic evaluation. Old aged WT mice were used as control. The mouse blood was collected by cardiocentesis immediately after euthanasia by CO_2_ inhalation. Haematology was performed on blood samples anticoagulated with EDTA using commercially supplied tubes (Microvette 100, Sarstedt AG, Numbrecht, Germany). The complete blood count was performed using the Hemavet 1700 Veterinary Multispecies Haematology System (Drew Scientific, Miami Lakes, FL). Blood smears of each sample were prepared, dried, fixed in methanol, and stained using Wright-Giemsa stain for microscopic evaluation. Serum measurements of blood urea nitrogen (BUN), creatinine, alanine aminotransferase (ALT), aspartate aminotransferase (AST), alkaline phosphatase, and total protein (TP) were determined using the Liasys 330 liquid reagent chemistry analyser (AMS Diagnostics, Weston, FL). After weighing the mice, kidneys and livers were harvested and fixed in 10% neutral buffered formalin. Following fixation, the tissues were trimmed, paraffin-embedded, and prepared as 5-micron sections and then stained with haematoxylin and eosin for standard histopathologic evaluation.

**Co-culture experiment followed by periodic imaging using Incucyte imager**

OT--NG CD8^+^ T cells were isolated from the spleens of Nur77-GFP; OT-I double transgenic mice using mouse CD8a^+^ T Cell Isolation Kit (Miltenyi cat. #130-095-236) as per the manufacturers’ protocol. 96-well plates were coated with 1 µg/mL for aCD3 for overnight at 4°C has used for plating T cells with 5 µg/mL aCD28 and IL2. Post 18 hours, plates were aspirated off antibodies, washed twice with PBS and plated with isolated CD8^+^ T for 12 hours using RPMI-1640 medium containing 10% FBS, 1% antibiotics, L-Glutamine 2 mM, and 2-mercaptoethanol 0.05 mM.

For T-cell cytotoxicity assay, the target KPPC-Ova cells were stained with DiR lipophilic tracer (Lumiprobe cat. #3433) for 15 min and seeded into 48-well flat bottom plate (4000 cells/well) with complete medium. In parallel BMDM post differentiation and treatment from C and CEG treated were prepared and added onto tumour cells during coculture. Post T & M cells coculture, T cell was added followed by Incucyte® Annexin V Red Dye (cat. #4759) where CD8^+^ T cells were added at the cancer cells and M cells coculture at ratio of 1:1:1. After 30-minute settle down at ambient temperature, plates were placed into the Incucyte live cell analysis instrument following scans every 3 hours. Five cell culture replicates were performed for each condition.

**Adoptive macrophage transfer**

To deplete tissue-resident macrophages, 8-10-week-old C57BL/6 (B6) mice were treated with

three doses of CSF1 neutralizing antibody (1 mg, 0.5 mg, 0.5 mg on days 3, 10, and 17) and three doses of clodronate-containing liposomes (200 μL on days 4, 11, and 18). Control mice received IgG and control liposomes (or PBS). Macrophage depletion was confirmed via FACS. Differentiated WT or *Ccrl2* KO macrophages were co-injected with tumour cells at a 1:1 ratio (50,000 cells of specified genotype). After 7 and 14 days, mice were imaged to observe macrophage and tumour distribution. Seven days post-implantation, mice were randomly assigned to each treatment group. Both groups received vehicle or C-E-G treatment twice a week via IV injection. Tumours were collected for weight and immune cell profiling by multiplex IHC, FACS or qRT-PCR.

**Single-Cell RNA sequencing and analysis**

Single-cell RNA sequencing was performed on PDAC tissues from vehicle- and drug-treated KPPC tumours at 14 days post-treatment. Each sample was pooled from 4 mice per group. Live cells were encapsulated using Chromium Single Cell 5′v2 kits (10× Genomics), and libraries were sequenced on a NovaSeq 6000 (Illumina) with an average of 50,000 reads per cell. Cell ranger was used for demultiplexing and alignment to the mm10 reference. Seurat was used for quality control, excluding cells with <1,000 or >6,000 genes or >10% mitochondrial RNA. SC Transform normalized and scaled data, regressing out cell cycle effects. The top 3,000 variable features were used for multi-set canonical correlation analysis, followed by Harmony integration. Dimensionality reduction was performed using PCA and UMAP, with clustering via shared nearest neighbour optimization. Differentially expressed genes (DEGs) were identified using MAST and filtered (p < 0.05), followed by Gene Set Enrichment Analysis (GSEA) for pathway analysis (GO, KEGG, Reactome, MSigDB).

**TCGA Database Analysis**

*CCR2* and *CCL2* expression, correlation was obtained and analyzed using GEPIA2 website.

**Supplementary Figure 1 (Supporting Figure 1)**

Bar graphs showing (**a**) expression of *CCR2* mRNA in PDAC vs normal pancreatic tissue and (**b**) summarizing relative expression of *CCR2* among different cancer types based on TCGA PDAC database. (**c**) Violin plot showing relative expression of *CCR2* mRNA among different clinical stages of PDAC types based on TCGA PDAC database. Bar graphs summarizing (**d**) relative expression of *CCL2* or (**e**) Pearson correlation coefficient between *CCR2* and *CCL2* among different cancer types based on TCGA PDAC database. (**f**) qRT-PCR showing diminished expression of *Ccr2* mRNA in murine KPPC cells stably expression empty vector (EV) or Cas9 and different small guide RNAs against *Ccr2*. KPPC cells were lentiviral infected with three different sg*Ccr2* sequences were tried, and two different pools for each sg*Ccr2* sequence were generated and tested for gene expression. Clone sg#2A and 2B were pooled and used for experiments.


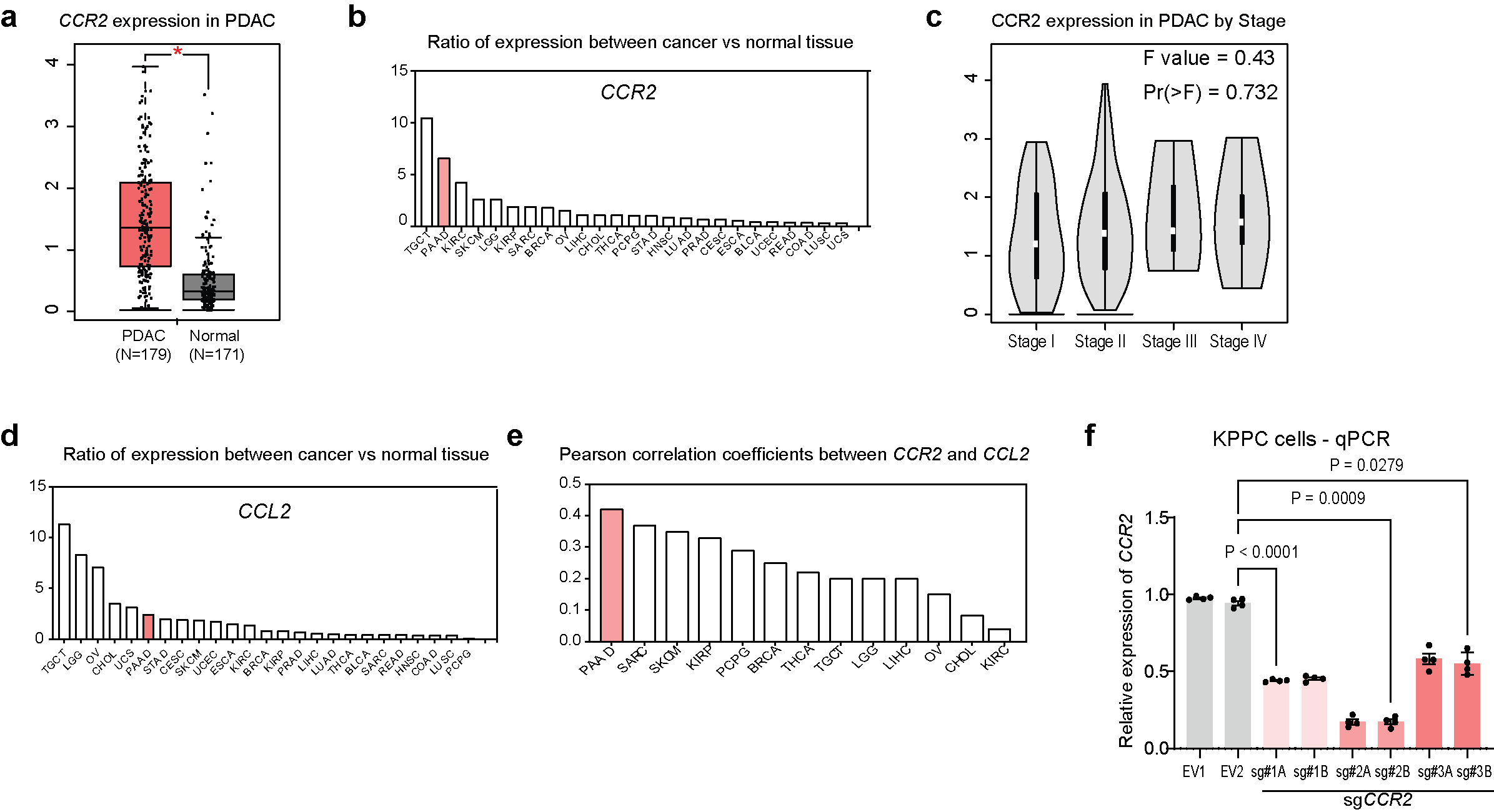


**Supplementary Figure 2 (Supporting Figure 2)**

**(a**) PET images and quantification of ^64^Cu-C or ^64^Cu-C-E-G signals in the PDAC of autochthonous KPPC mice at 6 weeks of age when PDAC starts to develop. (**b**) Representative multi-IF images and quantification of CK19 (PDAC cells) or F4/80 (TAMs) cells expressing CCR2 in KPPC PDAC tumours. 6tumours/arm and 4-5 fields per tumour were analyzed and quantified using HALO software. Representative IHC images and quantification of (**c**) aSMA or (**d**) Sirius red area in KPPC PDAC tumours treated as indicated for two weeks. 5 tumours/arm and 4-5 fields per tumour were analyzed and quantified using HALO software. (**e**) Bar graph showing relative viability by Alamar blue assay of fibroblasts derived from KPPC tumours, macrophages and KPPC cells treated as indicated for 36 hours. P values were calculated by two-tailed unpaired t test or two-way ANOVA followed by Tukey’s multiple comparison test.


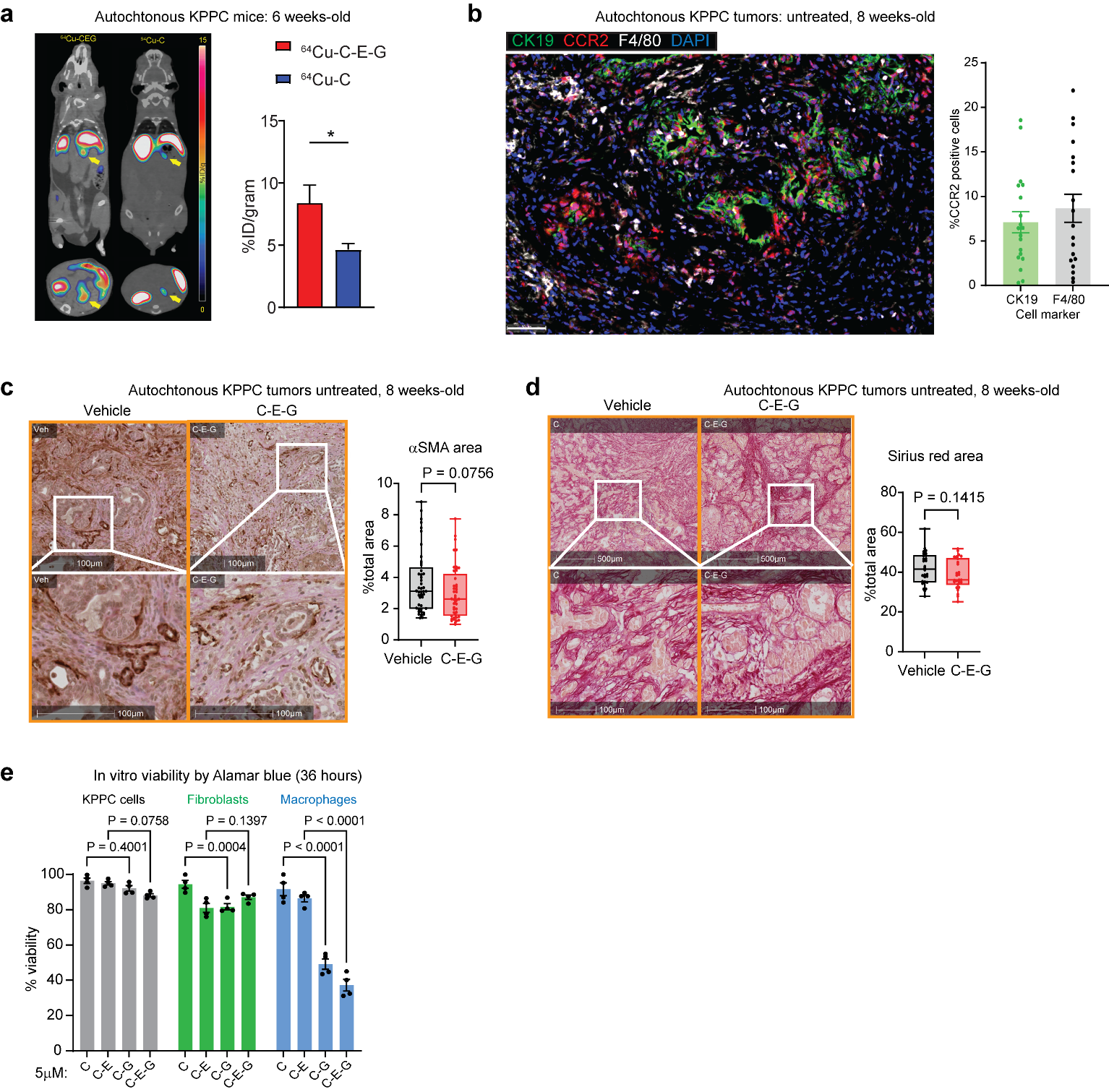


**Supplementary Figure 3 (Supporting Figure 3)**

(**a**) Heatmap showing differentially expressed genesets (DEGs) used to define different cellular subsets in the analysis of the KPPC scRNAseq data. Top 10 marker genes of each cluster were presented. (**b**) Violin plots showing single-cell RNA-sequencing–based expression levels of significantly upregulated cytokines and chemokines in TAMs from vehicle- and C-E-G–treated orthotopic KPPC tumours. Expression of *Cxcl2, Cxcl10, Il1b, Tnf,* and *Vegfa* are shown, with statistical significance indicated above each comparison. (**c**) Proposed TNF-related interaction emanating from TAMs to other cell types nominated by Cellchat analysis of C-E-G-treated KPPC scRNAseq data. (**d**) Dot plot showing enriched or diminished Hallmark GSEA signatures in the fibroblasts of C-E-G-treated KPPC tumours.


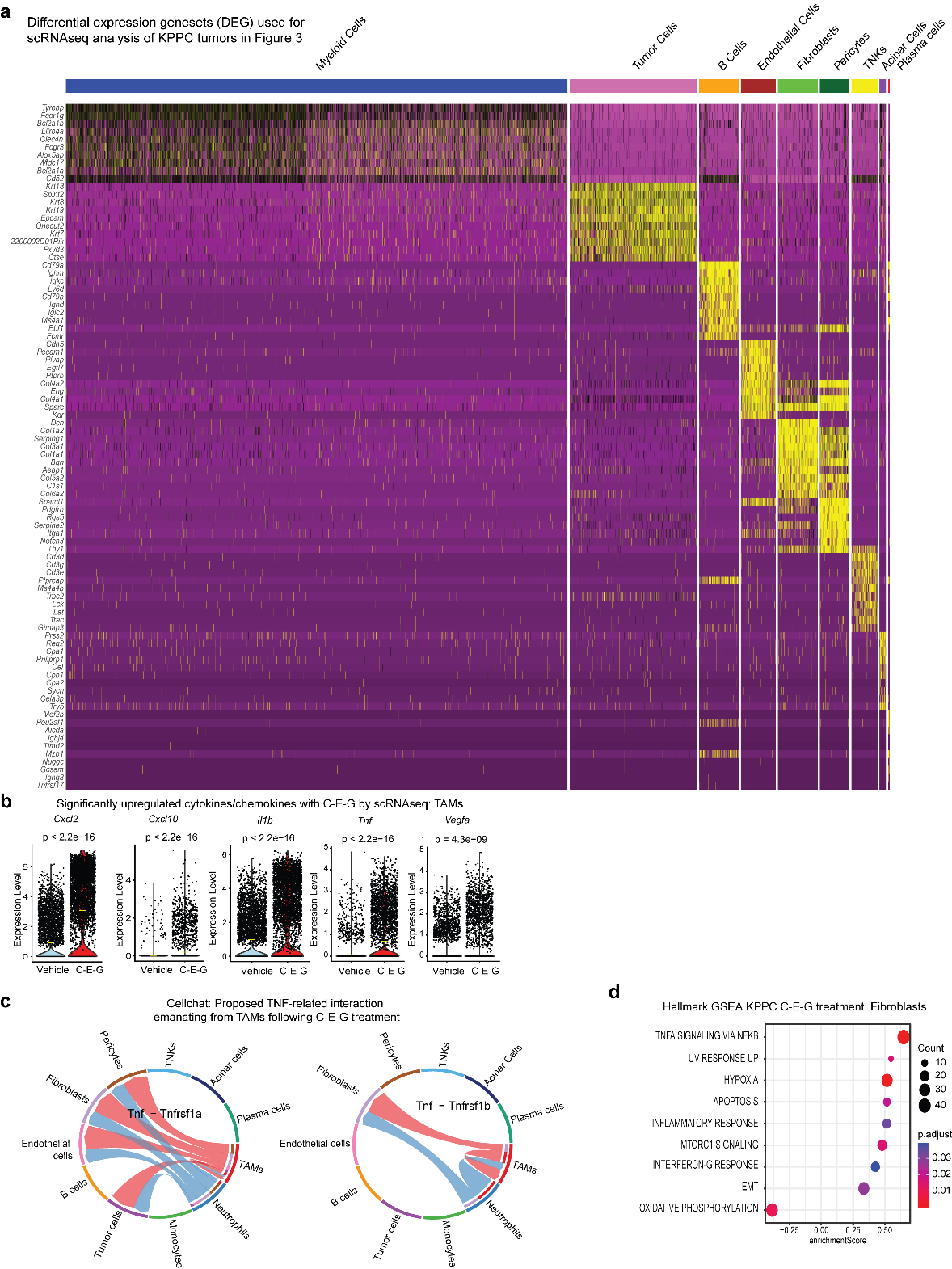


**Supplementary Figure 4 (Supporting Figure 4)**

(**a**) UMAP visualization of CCR2 expression distribution across all tumour-associated cell populations in KPPC tumours from vehicle-treated (control) and C-E-G–treated mice. Cells are colored by normalized CCR2 expression level, demonstrating the cellular sources and treatment-associated modulation of CCR2 expression within the tumour microenvironment. (**b**) Single-cell RNA sequencing analysis showing CCR2 expression across major cellular compartments in autochthonous KPPC tumours collected 14 days after treatment initiation in 6-week-old mice. Violin plots depict CCR2 expression levels in tumour-associated macrophages (TAMs), cancer-associated fibroblasts (CAFs), monocytes, dendritic cells (DCs), NKT cells, NK cells, CD8⁺ memory T cells, and plasma B cells under vehicle or C-E-G treatment. Statistical comparisons between treatment groups are indicated. (**c**) Dot plot shows upregulated expression of the indicated genes involved in antigen presentation in vehicle or C-E-G-treated KPPC tumours based on scRNAseq data.


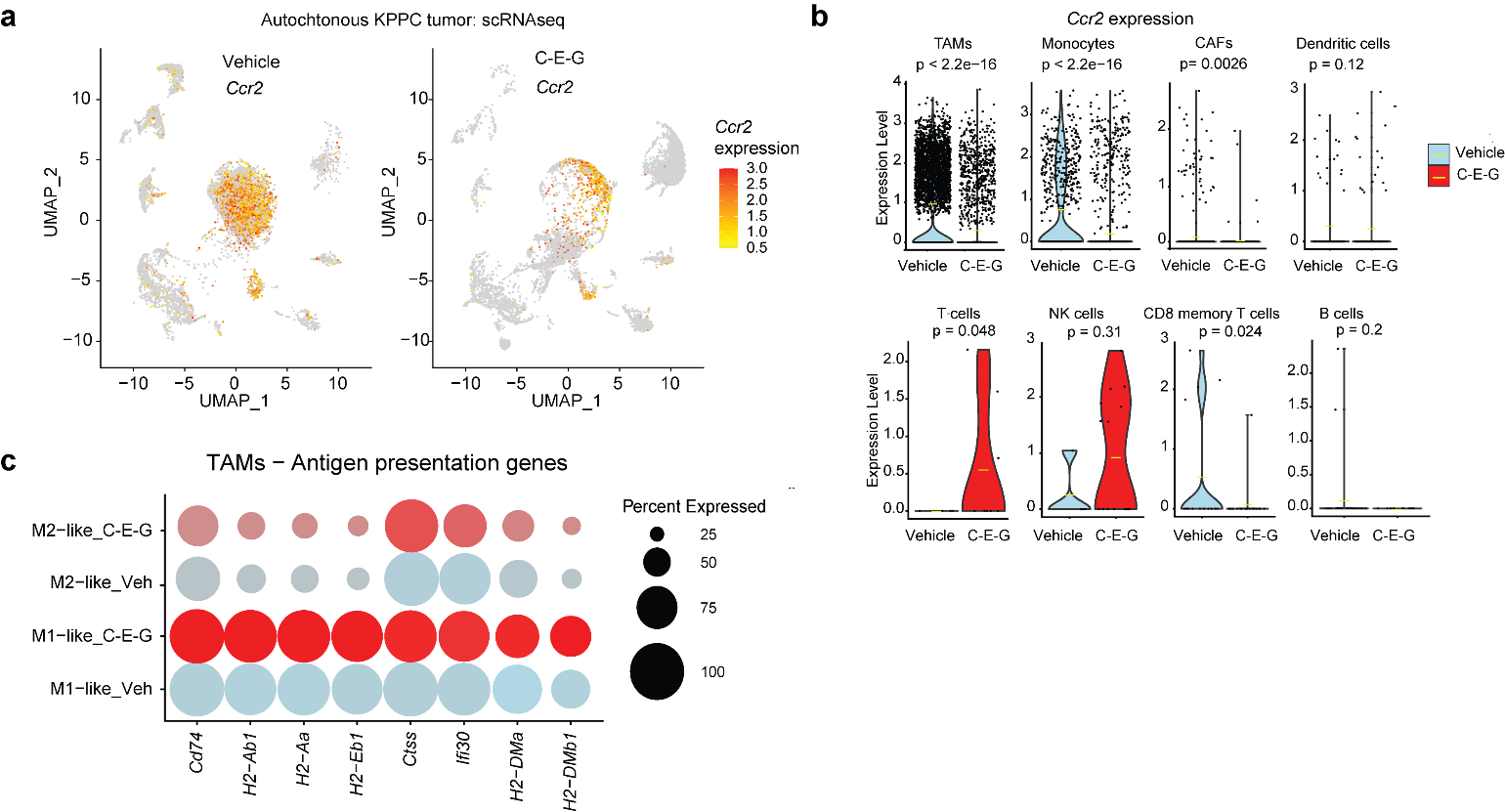


**Supplementary Figure 5 (Supporting Figure 5)** CCRL2-associated immune activation and macrophage-dependent T cell function. (**a**) Kaplan–Meier overall survival analyses of patients with PDAC stratified by CCRL2 expression using GEPIA2. Survival curves are shown for basal subtype, classical subtype, and combined basal and classical cohorts. Patients were divided into high and low CCRL2 expression groups based on median expression. Log-rank P values, hazard ratios (HR), and sample sizes are indicated for each cohort. (**b**) Scatter plot showing the correlation between CCR2 and CCRL2 mRNA expression in PDAC samples from the TCGA database. Expression values are shown as log2-transformed TPM. Pearson correlation coefficient (R) and corresponding P value are indicated. (**c**) Schematic illustrating isolation of murine BMDMs from WT or *Ccrl2^-/-^* C57BL/6J mice, differentiation into macrophages followed by co-culture with freshly isolated splenic T cells in the presence of absence of conditioned media from KPPC cells. Two days later, T cells and macrophages were subject to FACS. Bar graphs showing relative expression of (**d**) IFNg and CD107 on T cells cocultured with WT or *CcrlL2^-/-^* macrophages and (**e**) MHC II and CCR2 of WT or *CcrlL2^-/-^* macrophages in the co-cultures. (**f**) Schematic and representative images showing the co-culture of KPPC-ova or OT-NG CD8^+^T alone or in combinations with C or C-E-G-treated macrophages. KPPC-ova cells were pre-stained with Lumiprobe® DiR lipophilic tracer, a near-infrared fluorescent carbocyanine dye for cell membrane labelling prior to co-culture, and Annexin V NIR red dye was used to discern apoptotic cells. GFP fluorescence emanating from OT-NG CD8^+^ T cells indicates T cell activation. (**g**) Serial quantification of GFP positive (indicative of activated state) of OT-NG CD8^+^ T cells cultured alone or with the indicated cell types over time. (**h**) Serial quantification of Annexin V+ (indicative of apoptosis) KPPC-ova cells cultured alone or with the indicated cell types over time. P values were calculated by two-tailed unpaired t test or two-way ANOVA followed by Tukey’s multiple comparison test. FACS-based quantification of the indicated intratumoural (**i**) total immune CD45^+^ and CD11b^+^ cells, as well as (**j**) CD3^+^ and CD4^+^ T cells from the indicated transplanted KPPC tumours. (**k**) Flow cytometry–based quantification of CCRL2⁺ TAM populations in orthotopic KPPC tumours established in 8-week-old WT or CCR2-deficient (CCR2 KO) host mice. Left, schematic of experimental design. Right, bar graphs show the frequency of total CCRL2⁺ TAMs, monocyte-derived (Tim4⁻) CCRL2⁺ TAMs, and tissue-resident (Tim4⁺) CCRL2⁺ TAMs following vehicle or C-E-G treatment. (**l**) Tumour weight and Flow cytometry analysis of CCRL2⁺ TAM abundance in orthotopic KPPC tumours grown in WT or CCR2 KO host mice after vehicle or C-E-G treatment. Bar graphs summarize tumour weight (mg) and the percentage of CCRL2⁺ TAMs among total TAMs, with individual data points representing biological replicates and P values indicated.


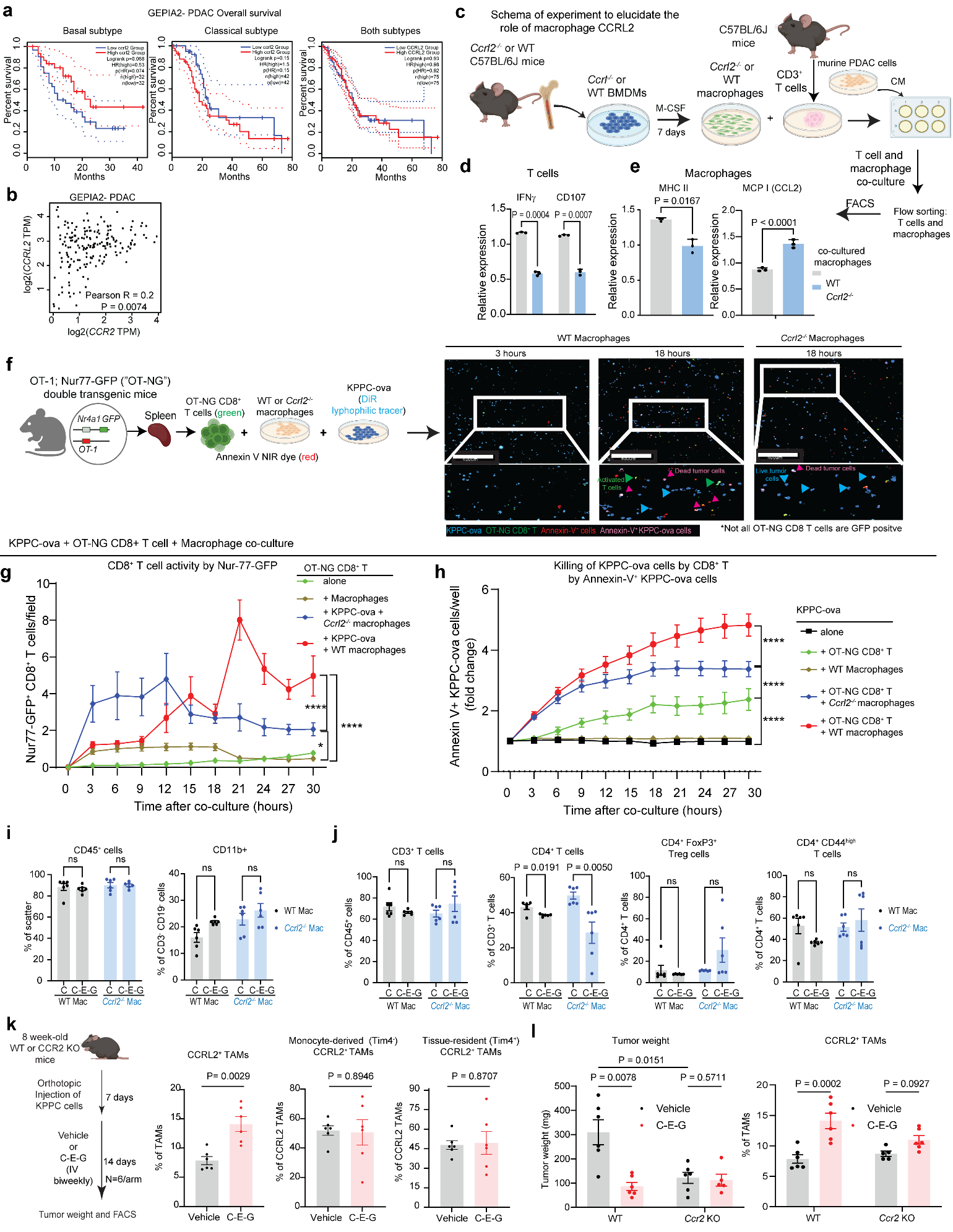


**Supplementary Figure 6 (Supporting Figure 6)**

(**a**) Experimental schema and final tumour weight of KPPC cells stably expressing chemerin or EV that were subcutaneously inoculated into C57/BL/6J mice followed by treatment with C or C-E-G for two weeks. (**b**) PET images and quantification of ^64^Cu- C or ^64^Cu-C-E-G signals in the subcutaneous KI tumours in live tumour-bearing mice. Right lower corners: ex vivo autoradiography image of KI tumours harvested 24 hours after mice were treated with ^64^Cu-C-E-G or ^64^Cu-C. Bar graphs comparing (**c**) blood counts and hemogram d) blood chemistries including liver and kidney function and (**e**) whole body weight and weights of the indicated organs harvested from KI-bearing mice treated with vehicle or C-E-G until euthanasia. (**f**) Schematic of the orthotopic KPPC tumour model and treatment regimen in C57BL/6J mice. KPPC cells were orthotopically injected into the pancreas, and mice were randomized 7 days later to receive vehicle, C-G, or C-E-G for 14 days. Tumours were harvested at the experimental endpoint for measurement of tumour weight. Flow cytometry-based quantification (**g**) of total monocytes (CD11b⁺Ly6G⁻Ly6C⁺) across compartments. Left panel shows the distribution of total monocytes in tumour, PB, and BM. Middle and right panels show the percentage of total monocytes in BM, PB, and TME following vehicle or C-E-G treatment. Each dot represents one mouse. (**h**) Analysis of CCR2⁺ monocytes across compartments. Bar graphs show the percentage of CCR2⁺ monocytes within total monocytes in tumour, PB, and BM, and the proportion of CCR2⁺ monocytes in the TME following vehicle or C-E-G treatment. Individual dots indicate biological replicates. (**i**) Assessment of apoptotic CCR2⁺ monocytes and TAMs. Bar graphs show the percentage of apoptotic CCR2⁺ monocytes in BM, PB, and TME, and apoptotic CCR2⁺ TAMs in PB following vehicle or C-E-G treatment. Apoptosis was assessed by Annexin V–based flow cytometry. (**j**) Evaluation of CCR2 expression in non-myeloid populations within the TME. Bar graphs show the percentage of CCR2⁺ EpCAM⁺ cells, CCR2⁺ fibroblasts, CCR2⁺ CD4⁺ T cells, and CCR2⁺ CD8⁺ T cells following vehicle or C-E-G treatment. (**k**) Dot plot showing upregulated expression of the indicated genes involved in T cell exhaustion in vehicle or C-E-G-treated KPPC tumours based on scRNAseq data.

**
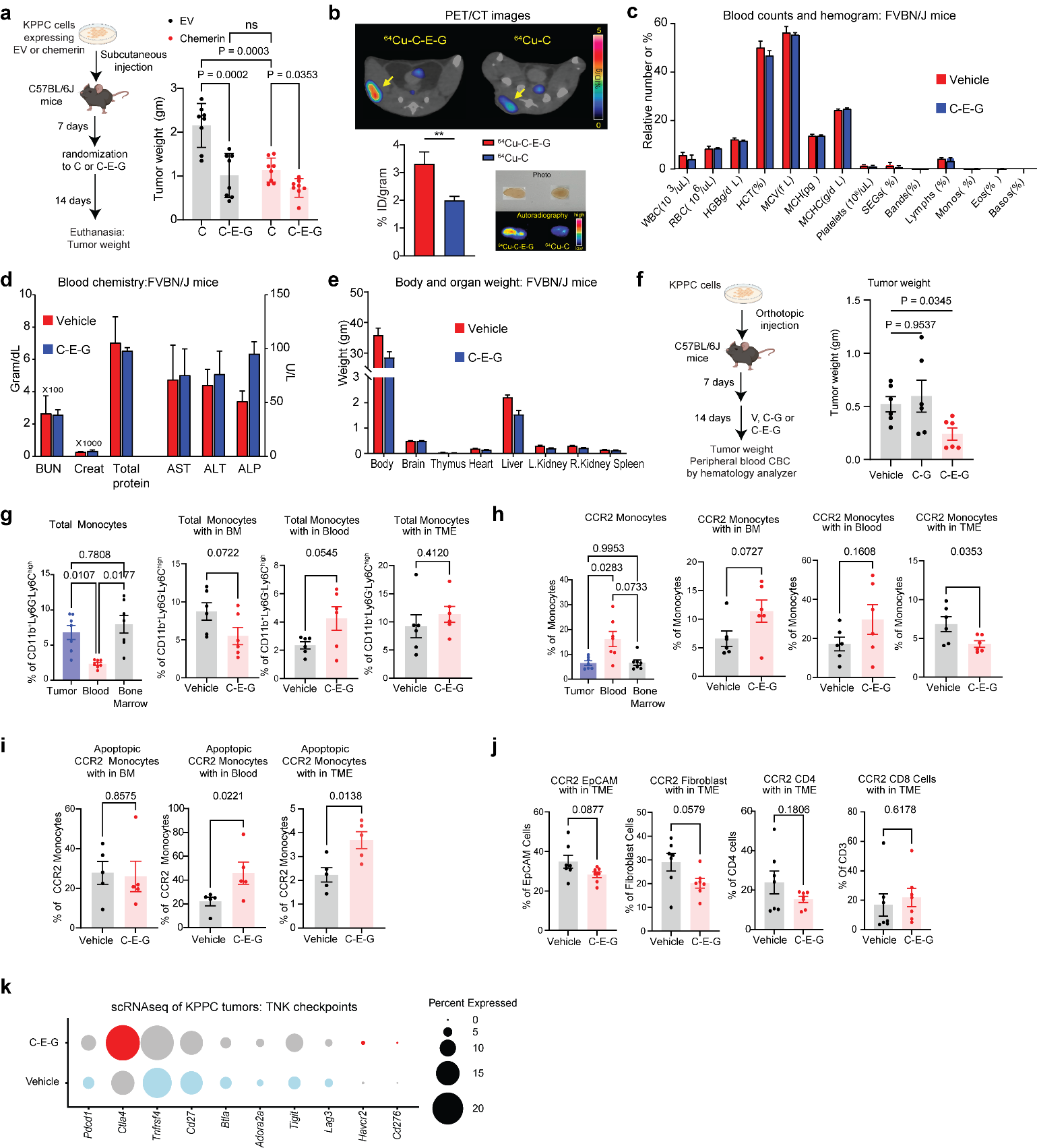
**

**REFERENCES**

1 Zhang, X. *et al.* Chemokine Receptor 2 Targeted PET/CT Imaging Distant Metastases in Pancreatic Ductal Adenocarcinoma. *ACS Pharmacol Transl Sci* **7**, 285-293, doi:10.1021/acsptsci.3c00303 (2024).

2 Zhang, X. *et al.* CC Chemokine Receptor 2-Targeting Copper Nanoparticles for Positron Emission Tomography-Guided Delivery of Gemcitabine for Pancreatic Ductal Adenocarcinoma. *ACS Nano* **15**, 1186-1198, doi:10.1021/acsnano.0c08185 (2021).
